# A systematic meta-review of interventions to prevent and manage delirium in the Intensive Care Unit: Part 2 – Non-pharmacological and multicomponent interventions

**DOI:** 10.1186/s13054-025-05726-8

**Published:** 2025-11-21

**Authors:** Burak Kundakci, Katherine L Jones, Andrew Booth, Louise Falzon, Maria Pufulete, Ben Gibbison

**Affiliations:** 1 School of Medicine and Population Health, University of Sheffield, Sheffield, UK; 2 The Centre for Musculoskeletal Research, University of Manchester, Manchester, UK; 3 Bristol Medical School, University of Bristol, Bristol, UK; 4 Department of Cardiac Anaesthesia and Intensive Care, University Hospitals Bristol and Weston NHS Foundation Trust, Bristol, UK

**Keywords:** Adult intensive and critical care, Delirium, Care-bundle, Care-package non-pharmacological intervention, Review of systematic reviews.

## Abstract

**Background:**

Intensive Care Unit (ICU) delirium is a multifactorial syndrome associated with prolonged hospitalisation, increased morbidity and mortality, cognitive decline, and higher healthcare costs. Prevention and management of delirium have to date included both pharmacological and non-pharmacological interventions. Many of these interventions have been combined in systematic reviews. This systematic meta-review (Part 2) reviews, details and analyses non-pharmacological and multicomponent interventions to prevent and manage ICU delirium.

**Methods:**

A comprehensive search was conducted across eight databases (MEDLINE, Embase, Cochrane Database of Systematic Reviews, Scopus, CINAHL, PsycINFO and Web of Science) from their inception until 8th August 2025. Eligible reviews included critically ill adults in ICU settings and examined the effectiveness of non-pharmacological and multi-component interventions on delirium occurrence, mortality, ICU and hospital length of stay (LOS) and other core outcomes. Reviews were assessed for quality using a framework of methodological interpretation of review conduct and reporting.

**Results:**

Thirty-two systematic review and meta-analyses including 335 single studies assessing 16 non-pharmacological interventions were included. The ABCDEF bundle and other multicomponent non-pharmacological interventions and early mobilization significantly reduced delirium incidence and duration. Family participation in care was also reported to reduce delirium incidence. Mortality benefits were reported in studies of multicomponent non-pharmacological interventions. The evidence was inconsistent and often of low quality.

**Conclusions:**

While some non-pharmacological interventions (multi-component care packages, early mobilization and family-based interventions) show potential to reduce delirium occurrence and duration, multicomponent strategies, particularly those including early mobilization and family participation, appear more effective.

**Supplementary Information:**

The online version contains supplementary material available at 10.1186/s13054-025-05726-8.

## Introduction

Delirium is an acute, fluctuating, and reversible syndrome resulting in impaired cognition and/or consciousness [1]. People with delirium have trouble maintaining attention and may have visual and auditory hallucinations [2]. While prevalence estimates vary, up to 7 in 10 patients admitted to intensive care units (ICUs) develop delirium, with estimates higher among mechanically ventilated patients [2, 3]. Patient risk factors for delirium include the severity of the acute illness, older age, and other comorbidities (cardiovascular risk factors, plus pre-existing impaired cognition, withdrawal from alcohol and nicotine) [4]. Risk factors relating to the management of critical illness include drugs (and dose) specific to the ICU e.g. sedatives and analgesia. The incidence of ICU delirium is expected to increase further with increasing admissions from an ageing population living with multiple long-term conditions.

ICU delirium is associated with a higher risk of mortality, morbidity, and prolonged need for mechanical ventilation, extending ICU and hospital lengths of stay and increasing healthcare costs [5–11]. There are also costs associated with longer-term sequelae, which may include dementia, other cognitive or functional decline, and increased need for long-term care [12, 13]. Multiple pharmacological and non-pharmacological interventions have been used to prevent and manage delirium in the ICU setting. Randomised controlled trials of interventions have been combined in multiple systematic reviews, meta-analyses, and network meta-analyses. However, there is a lack of overarching evidence syntheses and assessment of comprehensiveness to support future research and practice. We aimed to scope and map the meta-analysed evidence for interventions used for prevention and management of ICU delirium. This review is Part 2 of a pair of manuscripts detailing this work. (Part 1 details pharmacological interventions). The search strategy was common to both, but due to the different methods used for appraisal and analysis, and the vast quantity of data analysed, these 2 reviews are presented separately.

## Methods

The full protocol for this meta-review was published [14] and registered previously with PROSPERO (CRD42023473260). The evidence for pharmacological and non-pharmacological studies was conceptualised as a single meta review. However, given the volume of the evidence identified, we chose to present the pharmacological (Part 1) and non-pharmacological (including multi-component interventions Part 2) evidence separately.

We applied guidance from Cochrane overviews [15], the Preferred Reporting Items for Overviews of Reviews (PRIOR [16] and PRISMA Extension for Scoping Reviews (PRISMA-ScR [17] to support standardised evidence synthesis methods in the conduct and the reporting of the meta-review. We also sought input on aspects of the methodology from a Patient and Public Involvement group of people with lived experience of ICU delirium.

## Literature search

Our search included any type of pharmacological or non-pharmacological intervention and comprised a combination of subject headings and text words to represent the concepts of delirium AND intensive care AND systematic reviews. The search was performed in MEDLINE (Ovid), Embase (Elsevier), Cochrane Database of Systematic Reviews, Scopus, Cumulative Index to Nursing and Allied Health Literature (CINAHL), PsycINFO and Web of Science (from inception to 26 September 2023), as well as in initial scoping search of Epistemonikos (from inception to 19 July 2023), with manual deduplication of records. The search was subsequently updated in August 2025.

Although we pre-specified in our inclusion criteria that we would only include studies from the year 2000 onwards, most reviews we identified were published after this date, with only a minority of included trials published before 2000. Searches were unrestricted by language but limited to English at study selection. See Additional file 1 for full details.

## Eligibility

### Types of studies

We included systematic reviews and meta-analyses regardless of the country in which the primary research was conducted. No restrictions were applied based on the type of primary studies, such as RCTs or non-randomized designs. However, during the analysis, we identified systematic reviews with meta-analyses of RCTs when such evidence was available for individual interventions. Systematic reviews without meta-analyses were also examined if they included interventions that had not been evaluated in a meta-analysis.

### Type of participants

Critically ill adults (aged ≥ 18 years) were included. We identified ‘critically ill patients’ as those treated in a critical care or ICU of any specialty (e.g., burn, cardiac, medical, surgical, trauma) or high dependency unit (HDU). Patients with post-operative delirium were only considered eligible if they were in an ICU setting. We excluded studies of delirium related to alcohol withdrawal, as its underlying pathophysiology, clinical course, and management differ substantially from those of general ICU delirium [18]. We also excluded studies conducted in other intermediate care settings (e.g., coronary care units, respiratory high care units) due to differences in patient populations, care protocols, and environmental factors. Potentially relevant reviews including mixed settings (e.g., general hospital ward and ICU) were eligible for inclusion if ≥ 80% of included studies were reported to be conducted in the ICU.

### Types of interventions

Any non-pharmacological intervention and/or care bundle (also referred to as care package) or services interventions used in delirium prevention, treatment, or management compared to either each other, standard or usual care, and no treatment were included.

### Types of outcome measures

Outcomes were based on the Del-COrS core-outcome set [19] for research evaluating interventions to prevent or treat delirium in critically ill adults, as well as additional outcomes considered important by the research team and patient/carer partners.

We assessed outcomes separately for interventions aimed at: (i) preventing delirium, and (ii) treating or managing delirium.

#### Primary outcomes

For prevention, the primary outcome was delirium occurrence. The term “occurrence” is preferred over “incidence” in the Del-COrS core-outcome set [19], reflecting the fact that true delirium onset is unknown given the nature of critical illness (e.g. patients arriving to the ICU in a coma when delirium can’t be assessed). Although prevalence and incidence are sometimes used interchangeably in the literature, for this meta-review, both were categorized as delirium occurrence. If a review differentiated between these terms, it was documented.

For treatment or management, the primary outcomes were time to delirium resolution, duration of delirium (reported in days or hours), or delirium recurrence.

#### Secondary outcomes

- ICU length of stay (LOS) (days or hours as reported by the review).
- Hospital LOS (days or hours as reported by the review).
- Mortality (at any timepoint reported by the review).
- Time to delirium resolution or duration of delirium (at any timepoint reported by the review) (for prevention).
- Delirium severity (measured using any scale and timing, as reported by the review).
- Change in cognition including memory (measured using any cognitive scale and timing, as reported by the review).
- Change in emotional distress including anxiety, depression, acute stress, or post-traumatic stress disorder (using any symptom screening scale or diagnostic criteria at any timepoint reported by the review).
- Change in health-related quality of life (using any scale at any timepoint reported by the review.

### Classifying review interventions

An intensivist classified the interventions based on their nature. The classification highlighted overlapping categories, including circadian/sleep, family, physical/environmental, cognitive, and multi-component care bundle interventions.

Reviews were also classified as prevention, treatment or management, or unclear. The criteria for classification are shown in the data extraction template (Additional file 1). Classification was based on review-level reporting and included study descriptions.

### Study selection and data extraction

Search results were imported into Excel for screening after duplicates were removed in Endnote. Two of three reviewers (BK, KJ, AB) independently screened titles and abstracts, followed by full-text screening. Dual independent screening was conducted on at least 20% of records at both stages, based on meta-review eligibility criteria. A standardized data extraction form, developed by the review authors and pilot-tested on several systematic reviews, was used to capture demographics, methods, and results, as detailed in Additional File 1: Table S1. One reviewer (BK) extracted data, and a second member of the author team (BG) crosschecked a random 10% sample of included reviews for data entry with any discrepancies resolved by discussion.

### Assessing the quality of the review evidence

In accordance with published guidance for scoping and mapping-type reviews [20, 21], no formal critical appraisal or Grading of Recommendations, Assessment, Development and Evaluation (GRADE) was conducted to map the evidence. However, our overall assessment of review comprehensiveness involved methodological interpretation of review conduct and reporting, using standardized data extraction fields. Key factors considered in this assessment included: the use of network or pairwise meta-analysis, the review’s recency (published since 2019), the recency of included evidence (published since 2000), the number of included randomized controlled trials (RCTs), and the reporting of risk of bias assessments and GRADE (or equivalent quality criteria).

### Data analysis and synthesis

Evidence was presented based on the most recent, highest quality, most relevant, and most comprehensive meta-analysis. For each intervention, we first identified all relevant systematic reviews with meta-analyses. We then prioritized the most recent and comprehensive reviews. Among these, those that had conducted both risk of bias and GRADE assessments were selected as the primary source of evidence. If no such review was available, the most recent and comprehensive meta-analysis was used regardless of whether risk of bias or GRADE had been assessed. Interventions included in systematic reviews without meta-analyses were also reviewed to identify any interventions not assessed in meta-analyses. For interventions without meta-analysis data, it was aimed to include a corresponding systematic review. Where possible, quantitative evidence from the optimal review was reported narratively. No statistical analysis was performed.

## Results

### Search and study characteristics

**Fig. 1:**
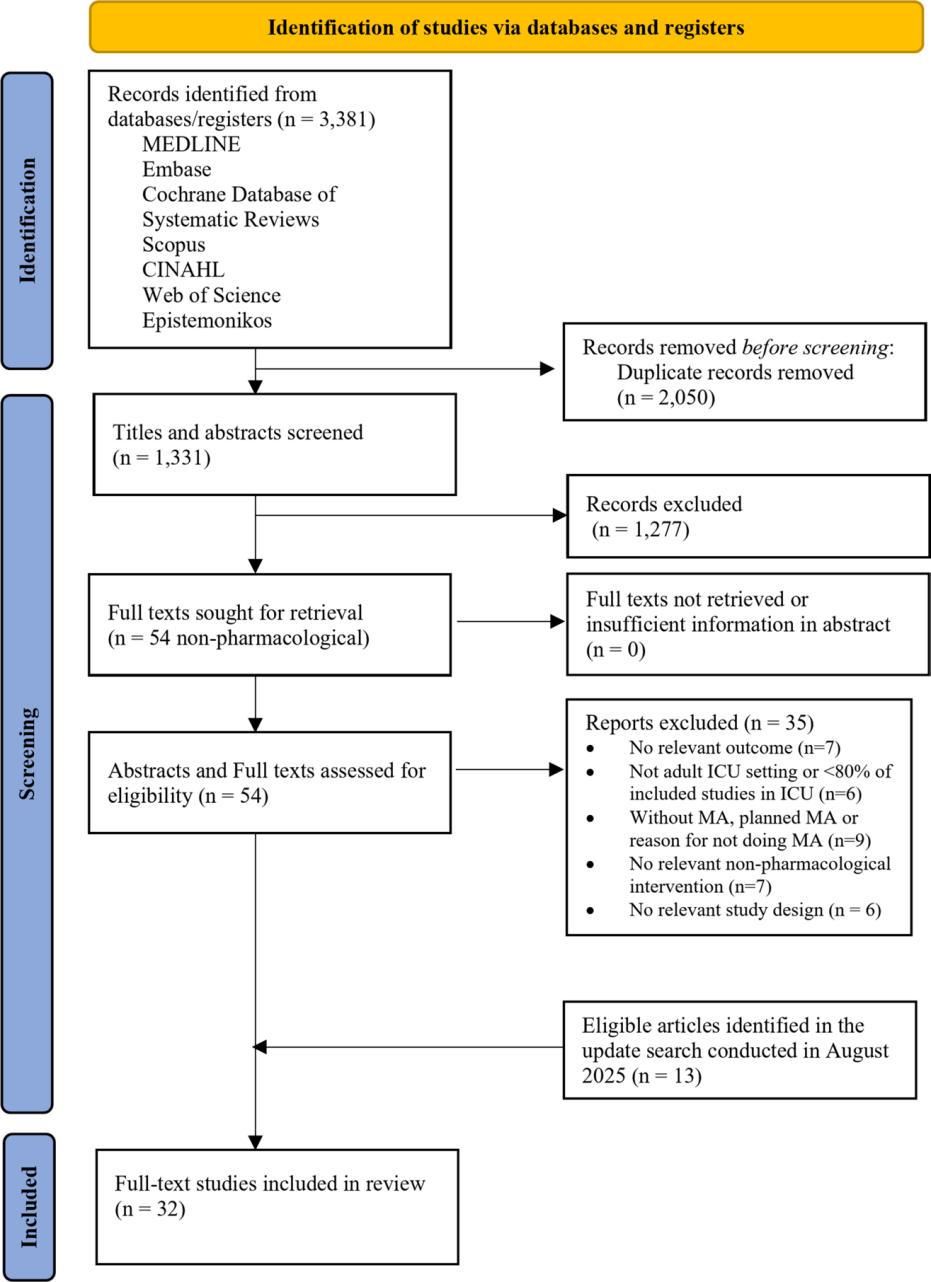
PRISMA flow diagram. Abbreviations. SR: systematic review; RCT: randomised controlled trial; ICU: intensive care unit; MA: meta-analysis*The list of excluded articles at the full-text screening stage, together with their exclusion reasons, was provided in the Additional file 1

Our search results are summarised in a PRISMA flow diagram (Fig. 1). Electronic database searches identified 3381 citations. After removing duplicates there were 1330 unique records. Title and abstract screening led to the exclusion of 1,276 studies. Fifty-four studies included eligible non-pharmacological interventions. We assessed the full texts of these, and 35 studies were excluded due to irrelevant outcome, missing meta-analysis, no relevant non-pharmacological intervention or mixed population. There were no interventions that were not evaluated by meta-analyses. The list of excluded articles at the full-text screening stage, together with their exclusion reasons, was provided in the Additional File 1: Exclusion reasons and references. The updated search identified 13 additional studies. Finally, 32 studies [22–53] met the inclusion criteria (Fig. 1).

The characteristics of the included systematic reviews with meta-analysis are summarized in Table 1. These studies were published between 2014 and 2025, with 25 (78%) published after 2020. There were 25 meta-analyses, two Cochrane systematic reviews with meta-analysis, four network meta-analyses (NMA), and one systematic review. Of these, 20 reviews (63%) included RCTs only, while 12 included both non-randomized studies and RCTs. While the number of primary studies within each review ranged from 6 to 118, the total number of relevant primary studies of non-pharmacological interventions relevant to this review varied from 1 to 59. Patient populations ranged from 49 to 36,356, although six studies did not report patient numbers. The primary studies were conducted across multiple countries, including the USA, China, Japan, UK, Korea, Türkiye, Italy, Chile, Brazil, Germany, Australia, Iran, Canada, Belgium, Netherlands, Thailand, Denmark, India, and Sweden. Funding sources indicated that 17 studies received public funding, eight reported no funding and seven did not report funding information.

**Table 1:** Characteristics of included studies

**Author** **year**	**Review type**	**Included study design(s)**	**N of included studies/N of included non-pharmacological RCTs**	**Countries**	**Inclusion criteria**	**N of participants**	**Review interventions**	**Outcomes**	**RoB**	**Quality/Certainty of the evidence assessment**	**Funding**	**Findings**
Al Qadheeb 2014	MA	RCTs	17/1	NR	adults (≥19 years or older) admitted to an ICU	49	Early mobilization after daily sedation interruption	Duration of delirium, short term mortality	Cochrane RoB2; LOW	NR	National Center for Advancing Translational Sciences, National Institutes of Health	A review of current evidence fails to support that ICU interventions that reduce delirium duration reduce short-term mortality. Larger controlled studies are needed to establish this relationship.
Bannon 2018	MA	RCTs	15/15	USA, Japan, Italy, Canada, Belgium, Netherlands, Chile, UK, Turkey, Thailand and Korea	Critically ill adult patients	2812	Physical and physical with occupational therapy; bright light therapy, range of motion exercises, earplugs, multicomponent orientation and cognitive stimulation protocol, multicomponent occupational therapy including positioning, cognitive training, relative involvement, a mirrors intervention, multicomponent targeting risk factors for delirium, reorientation using family voice	Incidence of delirium, duration of delirium, mortality, sleep quality, cognitive function, quality of life	Cochrane RoB2; LOW	Low quality of evidence	Public Health Agency	Current evidence does not support the use of non-pharmacological interventions in reducing incidenceand duration of delirium in critically ill patients. Future research should consider well-designed and well described multicomponent interventions and include adequately defined outcome measures.
Burry 2021	MA	RCTs	80/25	North America, Europe and Asia	critically ill adults (≥ 16 years of age in an ICU of any type or high-acuity unit)	NR	Multicomponent, environment physical therapy-occupational therapy	Delirium occurrence, mortality, ICU LOS, hospital LOS	Cochrane RoB2; LOW	NR	Canadian Institutes of Health Research	Single and multi-component non-pharmacologicalinterventions did not connect to any evidence networks to allow for ranking and comparisons as planned; pairwisecomparisons did not detect differences compared to standard care.
Chen 2022	NMA	RCTs	29/29	Brazil, Denmark, and US	adults (age ≥18 years) in ICU	6571	Light therapy, earplugs, combination of eye mask and earplugs, combination of eye mask, earplugs and melatonin, early mobilization, family visit, fluid management, reorientation, preoperative patient education and music	Incidence of delirium, duration of delirium	Cochrane RoB2; LOW	NR	Ministry of Science and Technology, Taiwan and Taipei Medical University Shuang Ho Hospital	Multicomponent strategy was the most effective non- pharmacological intervention in reducing the incidence of ICU delirium. Early mobilization and family participation involvement in non-pharmacological interventions seemed to be more effective in reducing the incidence of ICU delirium. These results of network-meta-analysis could be an important evidence-based for clinical healthcare providers to optimize the critical care protocol.
Dai 2025	MA	RCTs	14/14	Turkey, Iran, America, Korea, China, Netherlands	Patients aged 18 years or older receiving care in ICUs	1434	Music intervention	Risk of delirium	Cochrane RoB1; Overall risk of bias judgement by study = MODERATE	NR	Ministry of Science and Technology of Taiwan	Music interventions may reduce ICU delirium, particularly with two 30-minute daily sessions for 7 days, but low-certainty evidence warrants further high-quality research.
Fang 2025	MA	RCTs	18/18	China, India	ICU patients > 18 years of age	2717	Care bundles plus standard care	Incidence of delirium, delirium duration, hospital length of stay, mortality	Cochrane RoB1; Overall risk of bias judgement by study = HIGH	NR	None	Care bundles may reduce ICU delirium, but heterogeneity limits certainty; large multicenter RCTs are needed to identify optimal components.
Formenti 2023	SR	RCTs	12/12	NR	critically ill, adult patients in a medical or surgical ICU	NR	Acupuncture	Incidence of delirium	PEDro; Overall risk of bias judgement by study = MODERATE	NR	None	Acupuncture seems an effective part of a non-pharmacological strategy to manage agitation, pain and delirium in the ICU.
Herling 2019	MA (Cochrane)	RCTs	12/4	NR	adult ICU participants aged 18 years and above	1091	Physical or cognitive therapy or both, noise and light reduction in the ICU, and preventive nursing care	Delirium occurrence	Cochrane RoB2; LOW	Moderate to very low quality of evidence	National Institute on Aging	More research is needed to assess the effectiveness of physical and cognitive therapy in preventing ICU delirium, as well as the impact of sedation, environmental changes, and tailored nursing care.
Hu 2015	MA (Cochrane)	RCTs	30/3	China, Korea, Japan, Europe, and US	Critically ill adult patients with stable haemodynamic status whowere admitted to ICUs or critical care units and had a length of stay of more than 24 hours	NR	Earplugs and eyemasks, relaxation therapy, sleep-inducing music, massage, foot baths, aromatherapy, valerian acupressure, sound masking, and changingthe visiting times of family members	Incidence of delirium	Cochrane RoB2; LOW	Low or very low quality of evidence	School of Nursing, Fujian Medical University, China, National Natural Science Funds of China	Evidence on non-pharmacological sleep interventions in ICU adults is low to very low. Earplugs/eye masks may improve sleep and delirium incidence, but more high-quality research is needed.
Jarman 2023	MA	Mixed	12/10	Australia, USA, Iran, France, Turkey, Australia, Germany and UK	Participants aged over 18 admitted to ICU	1460	Physical activity intervention	Incidence of delirium, delirium prevalence, duration of delirium, delirium severity	Cochrane RoB2, PEDro scores and ROBINS-I; Overall risk of bias judgement by study = MODERATE TO HIGH	GRADE: Low quality of evidence	Health Education England (HEE)/National Institute for Health Research (NIHR) and the NIHR Oxford Biomedical Research Centre.	Evidence is insufficient to recommend physical activity alone for ICU delirium; intensity may matter, but more high-quality studies are needed.
Kang 2018	MA	Mixed	35/11	NR	Adult ICU patients (age ≥ 18 years)	NR	Multicomponent, physical environment, exercise, patient education, family participation	Incidence of delirium	EPOC; LOW	NR	Basic Science Program through the National Research Foundation of Korea (NRF) funded by the Ministry of Science and ICT	Multicomponent and physical environment interventions were the most widely used methods. Nonpharmacological interventions for the purpose of preventing ICU delirium were effective in reducing the duration of delirium and the delirium occurrence, but not the ICU length of stay and ICU mortality. None of the studies reported adverse reactions related to nonpharmacological interventions.
Kang 2023	MA	Mixed	118/59	NR	Adult patients admitted to the ICU	36356	Non-pharmacological interventions	Incidence of delirium, delirium duration	Cochrane RoB2, ROBIS-I; Overall risk of bias judgement by study = LOW TO HIGH	NR	Korea government and Ministry of Health & Welfare, Republic of Korea	Nonpharmacological interventions improve sleep and help prevent delirium in ICU patients. ICU nurses should adopt interventions that enhance person–environment compatibility to support better outcomes.
Li 2025a	NMA	RCTs	17/17	NR	Postoperative adult patients (≥18 years) in the ICU	1535	Sleep interventions	Incidence of delirium, ICU length of stay	Cochrane RoB2; Overall risk of bias judgement by study = MODERATE	CINeMA (Confidence in Network Meta-Analysis): Very low quality of evidence	NR	Our study shows that multicomponent non-pharmacological sleep interventions reduce delirium incidence and that circadian rhythm regulation improves sleep quality in postoperative ICU patients. ICU staff should prioritize these strategies to prevent delirium and enhance recovery.
Li 2025b	MA	RCTs	11/11	China, Brazil, Iran, Chile, Turkey, and Colombia	Critically ill patients without delirium (≥18 years old)	3113	Family involvement	Incidence of delirium, duration of delirium, ICU length of stay	Cochrane RoB2; Overall risk of bias judgement by study = MODERATE	GRADE: Very low to moderate quality of evidence	The National Key Research and Development Program of China	Family involvement plays a key role in preventing delirium in critically ill patients, with direct caregiving participation showing the greatest impact on reducing its incidence.
Liang 2021	MA	Mixed	34/10	Asia, US, UK, and Europe	ICU patients >18 years of age	7159	Multicomponent interventions,early mobilization, family participation, music, patient education, the physical environment,and sleep promotion	Incidence of delirium, duration of delirium, short term mortality, ICU LOS	Cochrane RoB2; LOW	Moderate to low/very low quality of evidence	None	Healthcare professionals should use single or multicomponent interventions (e.g., early mobilization, family participation) to prevent ICU delirium. Further research is needed on patient psychological and family outcomes.
Litton 2016	MA	Mixed	9/5	NR	Adult ICU patients	1455	Earplugs and co interventions such as eye mask, relaxing music	Duration of delirium, mortality	Cochrane RoB2; High	NR	NR	Earplugs in ICU patients, alone or with sleep hygiene measures, significantly reduce delirium risk, but the role of co-interventions and optimal sleep strategies remains unclear.
Lu 2019	MA	RCTs	13/3	NR	Patients were 18 years of age	94	Bright light	Incidence of delirium	Cochrane RoB2; HIGH	NR	Natural Science Foundation ofChina ((No. 81502182 and No. 81400893) and CPLA Youth TrainingProject for Medical Science, China (17QNP030)	Postoperative timed bright light exposure may be helpful for preventing delirium.
LV 2025	MA	RCTs	11/11	France, China, UK, Australia, Iran, Ireland	Adult patients admitted to ICU	3352	Patient and Family-Centered Care Interventions	Delirium prevalence, ICU length of stay, depression, anxiety	Cochrane RoB2; Overall risk of bias judgement by study = HIGH	GRADE: Low quality of evidence	NR	PFCC interventions may significantly reduce delirium rates among ICU patients; however, their effects on other outcomes, such as depression, anxiety, and length of stay, were not statistically significant.
Matsuura 2023	NMA	Mixed	11/3	Chile, UK, USA, China, Korea, Denmark	Patients admitted to the ICU aged 18 years and above	2549	Nonpharmacological multicomponent interventions	Incidence of delirium	Cochrane RoB2 and ROBINS-I; Overall risk of bias judgement by study = LOW to HIGH	NR	JSPS KAKENHI	Non-pharmacological interventions, especially multicomponent approaches (SP-CS-EM-PC-AS and SP-CS), were most effective in preventing delirium in critically ill patients.
Nydahl 2023	MA	RCTs	13/7	USA, Australia, Kore, Germany, Turkey, Chile, Japan	Critically ill adult patients	2164	Early mobilization	Delirium prevention, duration of delirium	JBI; LOW to MODERATE	Moderate quality of evidence	Not reported	The effect of mobilization on delirium is uncertain, though early mobilization may help prevent and shorten its duration. Further research is needed to determine the optimal approach.
Qin 2022	MA	Mixed	6/4	NR	Adult ICU patients	1825	Family intervention	Risk of delirium, duration of delirium, short term mortality, ICU LOS	Cochrane; LOW	Moderate quality of evidence	CAMS Innovation Fund forMedical Sciences (CIFMS)	Family intervention can reduce the risk of delirium in ICU patients. Further research is needed to identifyspecific family intervention therapies, how they are delivered,and their optimal duration.
Ryu 2022	MA	Mixed	11/1	NR	ICU patients aged over 19 years	NR	Preventing nursing intervention	Delirium occurrence, ICU LOS	ROBANS 2 for non-RCTs; HIGH	NR	Jungwon University Research Grant	For management of delirium among ICU patients, multi-component intervention packages, suitable for care setting in ICUs, need to be considered for the preparation of nursing intervention for prevention of delirium applicable to nursing practices.
Sosnowski 2023	MA	Mixed	18/2	USA, Korea, Italy, India, UK, Australia	Critically ill adult (age ≥ 18 years) patients	29576	ABCDE/ABCDEF bundle	Incidence of delirium, duration of delirium	JBI; LOW	Low quality of evidence	None	Data from eight studies showed that the ABCDE/F bundle significantly reduced delirium incidence and/or duration in adult ICU patients. However, high heterogeneity and low evidence certainty warrant cautious interpretation.
Soylemez 2023	MA	RCTs	14/8	USA, Brazil, UK, Iran, Turkey, Sweden	People with delirium hospitalised in ICU	1123	Multi-sensory stimulation,participation in activities of daily living and familyinvolvement, music therapy, ABCDE package, landscaping and family photo show	Duration of delirium	NR	NR	NR	Nonpharmacological interventions by nurses reduce ICU delirium duration, with effect size influenced by intervention type and study location. This study highlights their effectiveness in ICU care.
Teng 2023	MA	RCTs	15/8	NR	ICU patients (age ≥ 18 years)	NR	Sleep interventions (e.g., light therapy, earplugs, melatonin, and multicomponent nonpharmacologic treatments)	Incidence of delirium, ICU length of stay	Cochrane RoB2; Overall risk of bias judgement by study = High	NR	None	The current evidence suggests that non-pharmacological sleep interventions are not effective inpreventing delirium in ICU patients.
Wang 2020	MA	RCTs	39/5	Germany, China	adult (age >18 years) ICU patients	774	Early mobilization and rehabilitation (including a range of active or passive physical exercises, except for exclusively neuromuscular electrical stimulation, chest physical therapy, and Chinese medicine acupuncture)	Delirium rate	Cochrane RoB2; HIGH	NR	None	Evidence suggests early mobilization improves muscle strength, reduces ICU complications, and shortens mechanical ventilation and hospital stays. Its impact on delirium, mortality, and post-discharge quality of life requires further large-scale trials.
Xu 2022	MA	RCTs	7/7	NR	adult ICU patients with delirium	1291	Cognitive exercise	Incidence of delirium, duration of delirium, hospital LOS	Cochrane RoB2; MODERATE	NR	None	Meta-analysis confirms cognitive exercises shorten ICU delirium duration and hospital stay but do not affect incidence. The lack of post-discharge studies underscores the need for long-term research.
Xu 2025	MA	RCTs	14/14	USA, Japan, China, France and Korea	Critically ill patients aged 18 years or older	2151	Sensory-based intervention	Incidence of delirium	Cochrane RoB2; Overall risk of bias judgement by study = HIGH	GRADE: Moderate quality of evidence	Care Fund of the Second Hospital of Shanxi Medical University	Sensory-based interventions reduce delirium in critically ill patients, with auditory interventions showing the greatest benefit.
Zhang 2022	MA	Mixed	11/7	NR	Adult (18 years old or older) ICU patients	26384	ABCDEF bundle	Delirium prevalence, duration of delirium, mortality, ICU LOS, hospital LOS	Modified Jadad Scale and New Castle-Ottawa Scale; LOW	NR	NR	The meta-analysis found no evidence that bundle interventions reduce ICU delirium prevalence or duration but confirmed benefits in lowering coma days, hospital LOS, and 28-day mortality. Many studies did not fully address modifiable delirium risk factors, possibly limiting effectiveness. Rigorous RCTs and full ABCDEF bundle implementation are needed to assess their impact on delirium and related outcomes."
Zhao 2024	MA	RCTs	4/4	USA, Chile, Canada	Patients 18 years of age and older who were hospitalized for more than 24 h in the ICU	379	Occupational therapy	Incidence of delirium, duration of delirium, mortality, ICU and hospital length of stay	Cochrane RoB2; Overall risk of bias judgement by study = LOW to HIGH	NR	General Scientific Research Project of Zhejiang Education Department	While occupational therapy is less effective in preventing delirium, it significantly improves ADL and cognitive function in critically ill patients, making it a valuable part of multidisciplinary care.
Zhou 2025	MA	Mixed	17/7	NR	critically ill patients or those admitted to the ICU	1794	Early mobilization intervention	Incidence of delirium, duration of delirium, mortality, ICU and hospital length of stay	Cochrane RoB2 and ROBINS-I; Overall risk of bias judgement by study = LOW to MODERATE	NR	None	Early mobilization may reduce delirium risk and duration in critically ill patients and holds promise as a preventive strategy in ICU care.

ABCDEF, Assess, Prevent, and Manage Pain, Both Spontaneous Awakening Trials (SAT) and Spontaneous Breathing Trials (SBT), Choice of analgesia and sedation, Delirium: Assess, Prevent, and Manage, Early mobility and Exercise, and Family engagement and empowerment; ICU, Intensive Care Unit; LOS, Length of stay; MA, Meta-analysis; N, Number; NMA, Network Meta-analysis; NR, Not reported; RCT, Randomized controlled trial; RoB, Risk of bias

### Classifying review interventions

Fifteen studies clearly specified that the interventions were targeted at delirium prevention in the title or abstract. Twelve studies did not specify whether the interventions targeted prevention or treatment, while five studies addressed both treatment and prevention.

We identified five broad categories of interventions with a total of 16 specific intervention types. These included: [1] **Circadian/Sleep Interventions**, including bright light therapy, and earplugs and eye masks (or their combination); [2] **Family Interventions**, such as family participation interventions, family photo displays, and reorientation using family voice recordings; [3] **Physical/Environmental Interventions**, including acupuncture, early mobilization, fluid restrictions, massage therapy, mirrors intervention, music therapy, preoperative health education; [4] **Cognitive Interventions**, comprising cognitive exercises; and [5] **Multi-Component Care Bundles**, including the ABCDEF bundle, multicomponent non-pharmacological interventions, and preventive nursing care. Multicomponent non-pharmacological interventions refer to a multifaceted intervention involving a combination of several single-component non-pharmacological interventions in 1–4 above. The ABCDEF bundle stands for Assess, Prevent, and Manage Pain, Both Spontaneous Awakening Trials (SAT) and Spontaneous Breathing Trials (SBT), Choice of analgesia and sedation, Delirium: Assess, Prevent, and Manage, Early mobility and Exercise, and Family engagement and empowerment. The ABCDEF bundle was considered separately as it includes some pharmacological interventions, although the vast majority of its components are non-pharmacological.

### Assessment of risk of bias and quality/certainty of the evidence

The Modified Cochrane Risk of Bias tool (RoB2) or RoB1 was the most frequently used tool for assessing the methodological bias of primary studies, applied in 24 out of 32 reviews (75%). Other RoB tools included Newcastle-Ottawa, ROBINS-I, PEDro, JBI, EPOC, and Jadad. Most studies (if RoB1) or relevant outcomes (if RoB2) were judged to be at moderate to high RoB. Twenty reviews (63%) did not assess certainty of evidence using GRADE, but those that did rated the certainty of evidence as low to moderate. Most review authors cautioned that the findings should be interpreted carefully due to methodological limitations within the primary studies.

1. **Effect of non-pharmacological interventions on ICU delirium occurrence**

Overall, the evidence suggests that non-pharmacological interventions reduce the risk of delirium by about half (Table 2). Implementation of the ABCDEF bundle nearly halved delirium risk (Risk ratio (RR) = 0.57; 95% CI, 0.36 to 0.90) [35], while multicomponent interventions also significantly reduced occurrence (Odds ratio (OR) = 0.48; 95% CI, 0.34 to 0.69) [29]. Similarly, early mobilization was more effective than usual care in preventing ICU delirium (OR = 0.53; 95% CI, 0.34 to 0.83) [30]. For delirium treatment, early mobilization reduced the odds of delirium by just under 70% (OR = 0.33; 95%CI, 0.24 to 0.46, five studies, 859 participants) [29]. Family-based interventions such as extended visitation, active involvement, and family voice recordings reduced delirium risk by more than half (RR = 0.46; 95% CI, 0.31–0.69; very low– to moderate-certainty evidence) [46], although playing a recorded family voice alone was not effective (SMD = 0.28; 95% CI, − 0.60 to 1.16) [36].

2. **Effect of non-pharmacological interventions on duration of ICU delirium**

The evidence suggests that non-pharmacological interventions may reduce the duration of ICU delirium by between half to two days (Table 2). The ABCDEF bundle reduced delirium duration by around 1.4 days compared with usual care (MD = − 1.37; 95% CI, − 2.61 to − 0.13; low-certainty evidence) [35]. Multicomponent interventions similarly shortened delirium by about 1.5 days (MD = − 1.47; 95% CI, − 2.2 to − 0.75), although certainty was low and heterogeneity high (I² = 98%) [29]. Early mobilization demonstrated the most consistent effect, reducing delirium duration by nearly two days (MD = − 1.78; 95% CI, − 2.73 to − 0.83; moderate-certainty evidence, I² = 0%) [30]. Family interventions reduced delirium duration by just over two days (WMD = − 2.18; 95% CI, − 4.14 to − 0.22; three studies) [46], whereas approaches limited to photos or recorded voices did not show significant benefit (MD = − 0.71; 95% CI, − 2.54 to 1.12) [36].

3. **Effect of non-pharmacological interventions on mortality**

Multicomponent non-pharmacological interventions reported a potential mortality benefit across different timepoints within and post-ICU compared to control (OR = 0.51; 95% CI, 0.26 to 0.97) in two studies (419 participants) with no statistical heterogeneity (I² = 0%) [29]. No statistical difference was found for the ABCDEF bundle (RR = 1.01; 95% CI, 0.84 to 1.23) [39] or family interventions (OR = 0.68; 95% CI, 0.22 to 2.09) [36] for reducing ICU mortality.

4. **Effect of non-pharmacological interventions on ICU and hospital LOS**

Most reviews reported the effect of interventions on length of ICU stay. Overall, the evidence suggested that non-pharmacological interventions reduced ICU LOS by between one and two days (Table 2). The ABCDEF bundle reduced hospital LOS by around 1.5 days (MD = − 1.47; 95% CI, − 2.80 to − 0.15; five studies, 726 patients), although a larger meta-analysis of nine studies (5,184 patients) did not show a significant effect on ICU LOS (MD = − 1.08; 95% CI, − 2.16 to 0.00) [39]. Multicomponent non-pharmacological interventions were associated with a one-day reduction in ICU LOS (MD = − 1.01; 95% CI, − 1.77 to − 0.25; low-certainty evidence, I² = 70%) [29]. Family participation interventions reduced ICU LOS by about 1.5 days (MD = − 1.46; 95% CI, − 2.43 to − 0.50; seven studies) [46]. By contrast, early mobilization did not demonstrate a significant reduction (MD = − 1.02; 95% CI, − 2.88 to 0.84; two studies, 282 participants; very low-certainty evidence) [29].

**Table 2:** Effectiveness of non-pharmacological and multicomponent care bundle interventions for ICU delirium

**Intervention**	**ICU delirium occurrence**	**Duration of ICU delirium**	**Mortality**	**ICU and hospital LOS**
**Circadian/Sleep Interventions**				
Bright light therapy	OR 0.50 (95% CI 0.17 to 1.45); low RoB [45]	MD 0.02 (95% CI −1.15 to 1.19) [25]		
Earplugs and eye masks (or their combination)	OR 1.04 (95% CI 0.19 to 5.87); low RoB [45]			
**Family interventions**				
Family participation interventions	**OR 0.76 (95% CI, 0.67 to 0 86)**; six studies (1,825 participants); moderate certainty evidence; low to moderate RoB [46]	SMD 1.13 (95% CI −1.91 to 0.34); two studies [46]	OR 0.68 (95% CI 0.22 to 2.09); two studies [31]	**MD −2.31 (95% CI −4.14 to −0.48)**; three studies (833 participants) (ICU LOS) [46]
Landscaping and family photo displays		MD −0.71 (95% CI −2.54 to 1.12); two studies [36]		
Reorientation using family voice recordings	SMD 0.28 (95% CI −0.60 to 1.16); one study [36]			
**Physical/Environmental Interventions**				
Early mobilization	**OR 0.53 (95% CI 0.34 to 0.83)**; 13 studies (2,164 participants); moderate certainty of evidence; low to moderate RoB [30]	**MD −1.78 (95% CI −2.73 to −0.83)**; three studies (296 participants) [30]		MD −1.02 (95% CI −2.88 to 0.84); two studies (282 participants) (ICU LOS) [29]
Fluid management	OR 0.81 (95% CI 0.17 to 3.92); low RoB [25]			
Massage therapy	OR 0.76 (95% CI, 0.62 to 0.93); three studies (401 participants); moderate certainty evidence; high RoB [50]			
Mirrors intervention		**SMD −0.47 (95% CI −0.74 to −0.28)**; one study [36]		
Music therapy	**OR 0.47 (95% CI 0.28 to 0.79)**; two studies (311 participants); low certainty evidence, low RoB [41]			
Preoperative health education	OR 0.61 (95% CI 0.14 to 2.62); low RoB [25]			
**Cognitive Interventions**				
Cognitive exercises	OR 0.43 (95% CI 0.12 to 1.58); moderate RoB [38]	**MD −1.99 (95% CI −3.20 to −0.79) [**38]		**MD −2.10 (95% CI −2.48 to −1.72) **(hospital LOS) [38]
**Multi-Component Care Bundle**				
ABCDEF bundle	**RR 0.57 (95% CI 0.36 to 0.90)**; six studies (2,000 participants); low certainty evidence; low RoB [35]	**MD −1.37 (95% CI −2.61 to −0.13)**; five studies (3,418 participants) [35]	RR 1.01 (95% CI 0.84 to 1.23); (2,954 participants) [39]	MD −1.08 (95% CI −2.16 to 0.00); nine studies (5,184 participants) (ICU LOS) [39] **MD −1.47 (95% CI −2.80 to −0.15)**; five studies (726 participants) (hospital LOS) [39]
Multicomponent non-pharmacological interventions	**OR 0.48 (95% CI 0.34 to 0.69)**; 13 studies (3,172 participants); moderate to low/very low certainty evidence; low RoB [29]	**MD −1.47 (95% CI −2.2 to −0.75)**; seven studies (1,666 participants) [29]	**OR 0.51 (95% CI 0.26 to 0.97)**; two studies (419 participants) [29]	**MD −1.01 (95% CI −1.77 to −0.25)**; 10 studies (2,036 participants) (ICU LOS) [29]
Preventive nursing care	**RR 0.38 (95% CI 0.32 to 0.45); **16 studies; high RoB [42]	**WMD -1.60 (95% CI -1.96 to -1.23);** 9 studies [42]	RR 0.78 (95% CI 0.44 to 1.40); Three studies [42]	**WMD -3.19 (95% CI -4.19 to -2.18); **10 studies (Hospital LOS) [42]

ABCDEF, Assess, Prevent, and Manage Pain, Both Spontaneous Awakening Trials (SAT) and Spontaneous Breathing Trials (SBT), Choice of analgesia and sedation, Delirium: Assess, Prevent, and Manage, Early mobility and Exercise, and Family engagement and empowerment; CI, Confidence interval; ICU, Intensive Care Unit; LOS, Length of stay; MD, Mean difference; OR, Odds ratio; RoB, Risk of bias; RR, Risk ratio; SMD, Standardized mean difference. Text in bold shows significant values

## Discussion

This review of systematic reviews identified 32 systematic reviews and meta-analyses evaluating 16 non-pharmacological and multicomponent care bundle interventions for the prevention and management of ICU delirium. The evidence suggests that non-pharmacological interventions (particularly multicomponent approaches including the ABCDEF bundle, early mobilization, and family engagement) are associated with reductions in delirium incidence and duration, as well as some improvements in mortality and ICU/hospital length of stay (LOS). However, the certainty of evidence ranged from very low to moderate, and findings were inconsistent across some interventions.

Multicomponent non-pharmacological interventions (including the ABCDEF bundle) are the most promising approaches for delirium prevention and management, and this is consistent with previous research [54, 55]. The significant reductions in delirium occurrence and duration observed in our analysis suggest these bundled approaches are more effective than isolated interventions. This is because they target multiple delirium risk factors simultaneously. This approach aligns with the established multifactorial etiology of delirium and supports a holistic approach to patient care (even in the ICU) [56]. These results are further supported by a review using a different methodology (an umbrella review) [55] which synthesized 14 systematic reviews and/or meta-analyses, and similarly concluded that multicomponent non-pharmacological interventions are the most promising strategies for delirium prevention. These findings have also been reflected in published guidelines [54, 57] which recommend multicomponent non-pharmacological interventions to address modifiable risk factors. However, the specific components which need to be included, and ways of operationalizing and implementing them remains the biggest challenge in ICU delirium care. This has led to the difficulties in implementing any of the care bundles in routine clinical care [58, 59]. This is important because observational data from more than 15,000 ICU patients demonstrate a dose-response relationship: higher bundle compliance is linked to lower odds of delirium, coma, mechanical ventilation, and in-hospital death, as well as a greater likelihood of discharge [60].

There is considerable variation in how non-pharmacological interventions are defined, which makes it difficult to compare them in research and to implement them in practice. For example, early mobilization stands out as a particularly effective component among single interventions (consistent with previous research [54, 55]). However, the early mobilization interventions vary widely, ranging from passive range-of-motion exercises in bed using cycle ergometry to active out-of-bed mobilization. There were differences in the timing of initiation, the frequency of the intervention and who performed it (e.g. physiotherapists, nurses, occupational therapists, or mixed teams). Standardizing definitions of timing, content and duration would help teams design and deliver these interventions with greater fidelity.

The gap in understanding which specific components (and their timing or sequence) drive effectiveness within any multi-component intervention make designing the optimal care bundle difficult. A recent network meta-analysis by Chen et al. [25] compared five different types of multicomponent non-pharmacological interventions. They found that a multicomponent, non-pharmacological intervention comprising seven elements (including early mobilization and family participation) was found to significantly reduce the incidence of ICU delirium and demonstrated the highest efficacy. Although it might be assumed that incorporating additional components would enhance delirium prevention, this was not seen. Two explanations for this are possible: first, that implementation of more than seven components within a single care package is not feasible; or second, that only the seven included interventions are genuinely effective. The former explanation appears more plausible, given the behavioral and cultural challenges associated with implementing such care packages [68].

None of the multicomponent care packages used a behavioral framework in their design. In the complex setting of the ICU with competing priorities and limited resources [68]– this represents a significant issue in their implementation. The limited evidence for cost-effectiveness of any of these interventions despite the likelihood that these interventions affect ICU staff workload and, consequently, overall care costs also hinders implementation.

None of the interventions were powered for quality-of-life outcomes. Our Patient Participation and Inclusion Group consistently highlighted that as more people survive ICU, outcomes should focus on longer term, out of hospital, quality-of-life outcomes. This was highlighted in the James Lind Alliance Priority Setting Partnership for ICU – but still has not been implemented in the delirium setting [61].

This review has several strengths. A key strength is the inclusion of perspectives from diverse stakeholders, including patients, carers, intensivists, nurses, pharmacists, physiotherapists, psychologists and methodologists in the design and interpretation of non-pharmacological interventions for ICU delirium. By considering their experiences and preferences, we aimed to capture a more holistic view of the impact of these interventions. Furthermore, our review focused exclusively on meta-analyses of existing research. This approach allowed us to synthesize the highest level of available evidence, and it provided a more robust assessment of the effectiveness of non-pharmacological interventions for ICU delirium.

Our review also has some limitations. Firstly, the overall quality of the evidence was weak. Many of the included reviews highlighted low to moderate GRADE ratings and methodological limitations in the primary studies, such as small sample sizes, and lack of blinding due to the nature of the intervention. Secondly, while we aimed to include only review-level evidence from RCTs, this was not always possible. For some complex interventions, such as the ABCDEF bundle, pure RCT evidence was scarce. This required including studies with other designs, which may be more prone to bias and influence the strength of causal inferences. Thirdly, significant heterogeneity was observed across studies in terms of treatment protocols. Variations in the specific components of multicomponent interventions, the intensity and duration of interventions such as early mobilization, and the types of family involvement made it challenging to draw conclusions about specific interventions.

Future research should prioritise standardising intervention protocols and identifying effective implementation strategies. In particular, there is a pressing need for a well-designed randomised controlled trial (RCT) evaluating a multicomponent intervention that incorporates the most promising elements identified in this review, such as early mobility protocols, family engagement strategies, and targeted environmental modifications, alongside those highlighted in the complementary pharmacological review (Part 1 [69]). Such a rigorous evaluation would provide a stronger evidence base for clinical practice guidelines that is currently lacking.

**In conclusion**, while some non-pharmacological interventions (multi-component care packages, early mobilization and family-based interventions) show promise in reducing the duration and occurrence of delirium, the evidence is inconsistent and often of low quality. There was heterogeneity in the components that were included in the multicomponent interventions, as well as the definitions of the individual components themselves. Standardization of interventions as well as testing them in adequately powered RCT would be one of the most important ways to improve delirium prevention and care.

## Supplementary Information

Supplementary Material 1.

## Data Availability

The datasets used and/or analyzed during the current study are available from the corresponding author on reasonable request.

## Abbreviations

ABCDEF: Assess, Prevent, and Manage Pain, Both Spontaneous Awakening Trials (SAT) and Spontaneous Breathing Trials (SBT), Choice of analgesia and sedation, Delirium: Assess, Prevent, and Manage, Early mobility and Exercise, and Family engagement and empowerment
Del-COrS: Development of a core outcome set for effectiveness trials of interventions to prevent and/or treat delirium
GRADE: Grading of recommendations, assessment, development and evaluation
HDU: High dependency unit
ICU: Intensive care unit
JBI: Joanna Briggs institute
LOS: Length of stay
MA: Meta-analysis
MD: Mean difference
N: Number
NICE: The national institute for health and care excellence
NIMA: Network meta-analysis
NR: Not reported
OPTIC: OPTimising the prevention, identification and management of ICU-Delirium
OR: Odds ratio
PRISMA: Preferred reporting items for systematic reviews and meta-analyses
PRIOR: Preferred reporting items for overviews of reviews
RCT: Randomized controlled trial
RR: Risk ratio
RoB: Risk of bias
SCCM: The US society of critical care medicine
SMD: Standardized mean difference
