# Correction: A systematic meta-review of interventions to prevent and manage delirium in the Intensive Care Unit: Part 2 – Non-pharmacological and multicomponent interventions

**DOI:** 10.1186/s13054-025-05804-x

**Published:** 2026-02-11

**Authors:** Burak Kundakci, Katherine Jones, Andrew Booth, Louise Falzon, Maria Pufulete, Ben Gibbison

**Affiliations:** 1 School of Medicine and Population Health, University of Sheffield, Sheffield, UK; 2 Centre for Musculoskeletal Research, University of Manchester, Manchester, UK; 3 Bristol Medical School, University of Bristol, Bristol, UK; 4 Department of Cardiac Anaesthesia and Intensive Care, University Hospitals Bristol and Weston NHS Foundation Trust, Bristol, UK

**Correction: Critical Care (2025) 29:501**

10.1186/s13054-025-05726-8

Following publication of the original article [1], the authors identified errors in the affiliation of three authors, the institutional author list was incomplete in the HTML version, Fig. 1 was incorrect as it should read at Included ‘Full-text studies included in review (n = 32)’. Tables 1 and 2 weren’t the latest version. A reference (citation) to part 1 was not included. Both the incorrect and correct information is given hereafter.

Katherine Jones, Andrew Booth and Louise Falzon are affiliated with the University of Sheffield: 1School of Medicine and Population Health, University of Sheffield, Sheffield, UK.

The authors Burak Kundakci, Katherine L Jones, Andrew Booth, Louise Falzon, Maria Pufulete, Ben Gibbison that are also part of the institutional group the OPTIC consortium were missing in HTML version.

**The incorrect Abstract Conclusions section**:

**Conclusions** While some non-pharmacological interventions (multi-component care packages, early mobilization and family-based interventions) show potential to reduce delirium occurrence and duration. Multicomponent strategies, particularly those including early mobilization and family participation, appear more effective.

**The correct Abstract Conclusions section**:

**Conclusions** While some non-pharmacological interventions (multi-component care packages, early mobilization and family-based interventions) show potential to reduce delirium occurrence and duration, multicomponent strategies, particularly those including early mobilization and family participation, appear more effective.

The incorrect Fig. 1:


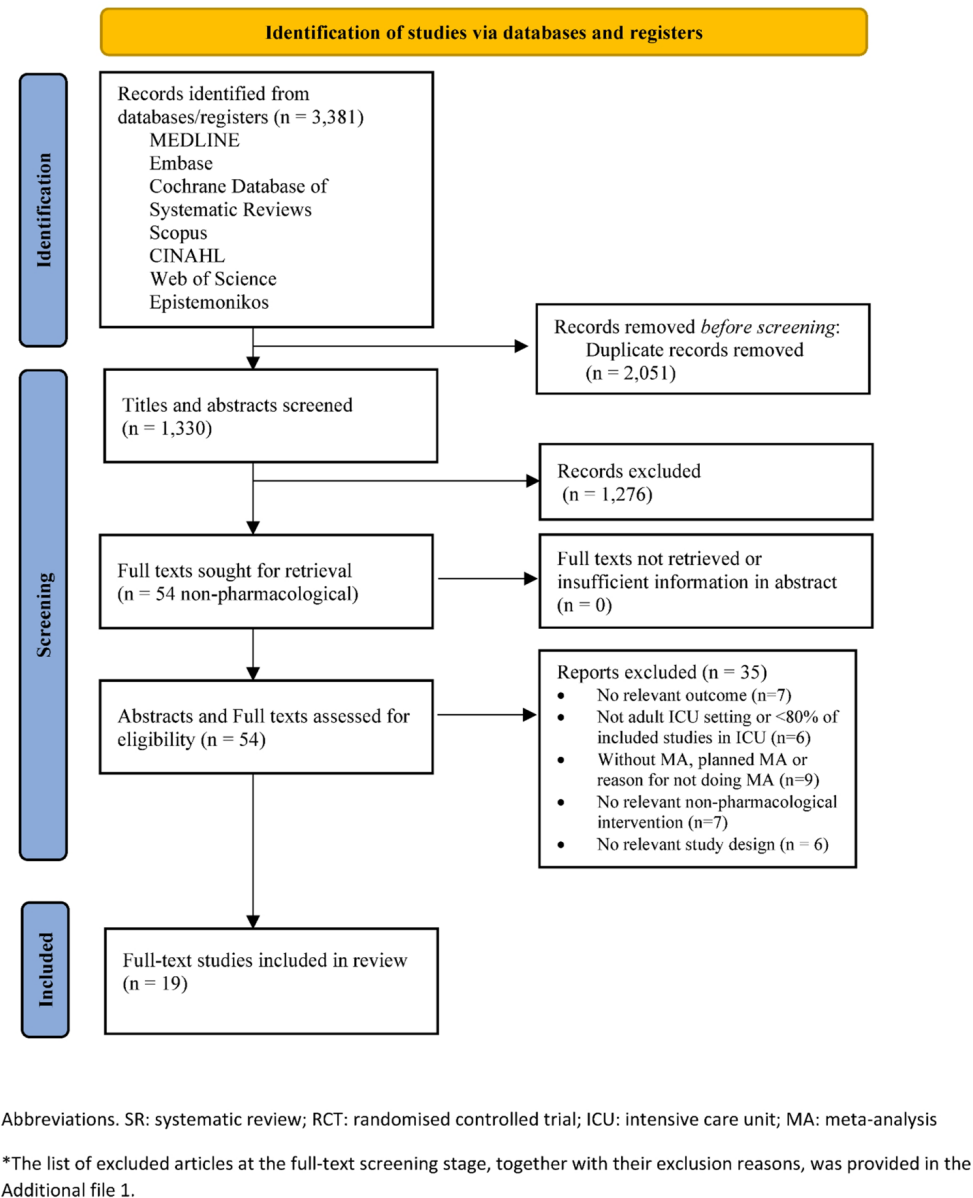


**Fig. 1** PRISMA flow diagram 

The correct Fig. 1:

**Fig. 1:**
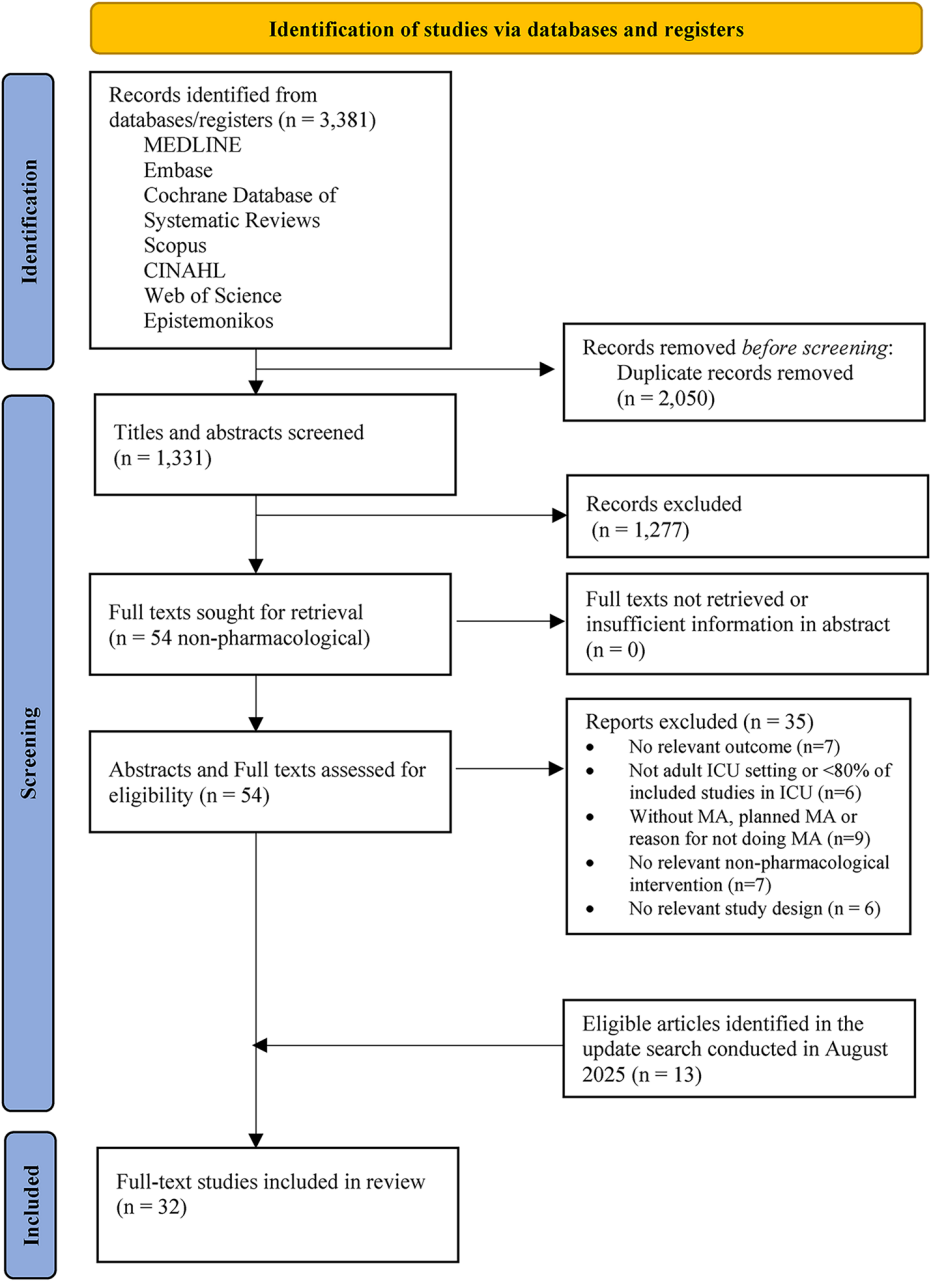
PRISMA flow diagram Abbreviations. SR: systematic review; RCT: randomised controlled trial; ICU: intensive care unit; MA: meta-analysis. *The list of excluded articles at the full-text screening stage, together with their exclusion reasons, was provided in the Additional file 1

The incorrect Table 1:

**Table:** **Table 1** Characteristics of included studies

Author year	Review type	Included study design(s)	*N* of included studies / *N* of included non-pharmacological RCTs	Countries	Inclusion criteria	*N* of participants	Review interventions	Outcomes	RoB	Quality/Certainty of the evidence assessment	Funding	Findings
Al Qadheeb 2014	MA	RCTs	17/1	NR	adults (≥ 19 years or older) admitted to an ICU	49	Early mobilization after daily sedation interruption	Duration of delirium, short term mortality	Cochrane RoB2; LOW	NR	National Center for Advancing Translational Sciences, National Institutes of Health	A review of current evidence fails to support that ICU interventions that reduce delirium duration reduce short-term mortality. Larger controlled studies are needed to establish this relationship
Bannon 2018	MA	RCTs	15/15	USA, Japan, Italy, Canada, Belgium, Netherlands, Chile, UK, Turkey, Thailand and Korea	Critically ill adult patients	2812	Physical and physical with occupational therapy; bright light therapy, range of motion exercises, earplugs, multicomponent orientation and cognitive stimulation protocol, multicomponent occupational therapy including positioning, cognitive training, relative involvement, a mirrors intervention, multicomponent targeting risk factors for delirium, reorientation using family voice	Incidence of delirium, duration of delirium, mortality, sleep quality, cognitive function, quality of life	Cochrane RoB2; LOW	Low quality of evidence	Public Health Agency	Current evidence does not support the use of non-pharmacological interventions in reducing incidence and duration of delirium in critically ill patients. Future research should consider well-designed and well described multicomponent interventions and include adequately defined outcome measures
Burry 2021	MA	RCTs	80/25	North America, Europe and Asia	critically ill adults (≥ 16 years of age in an ICU of any type or high-acuity unit)	NR	Multicomponent, environment physical therapy-occupational therapy	Delirium occurrence, mortality, ICU LOS, hospital LOS	Cochrane RoB2; LOW	NR	Canadian Institutes of Health Research	Single and multi-component non-pharmacological interventions did not connect to any evidence networks to allow for ranking and comparisons as planned; pairwise comparisons did not detect differences compared to standard care
Chen 2022	NMA	RCTs	29/29	Brazil, Denmark, and US	adults (age ≥ 18 years) in ICU	6571	Light therapy, earplugs, combination of eye mask and earplugs, combination of eye mask, earplugs and melatonin, early mobilization, family visit, reorientation, preoperative patient education and music	Incidence of delirium, duration of delirium	Cochrane RoB2; LOW	NR	Ministry of Science and Technology, Taiwan and Taipei Medical University Shuang Ho Hospital	Multicomponent strategy was the most effective non- pharmacological intervention in reducing the incidence of ICU delirium. Early mobilization and family participation involvement in non-pharmacological interventions seemed to be more effective in reducing the incidence of ICU delirium. These results of network-meta-analysis could be important evidence-based for clinical healthcare providers to optimize the critical care protocol
Dai 2025	MA	RCTs	14/14	Turkey, Iran, America, Korea, China, Netherlands	Patients aged 18 years or older receiving care in ICUs	1434	Music intervention	Risk of delirium	Cochrane RoB1; Overall risk of bias judgement by study = MODERATE	NR	Ministry of Science and Technology of Taiwan	Music interventions may reduce ICU delirium, particularly with two 30-minute daily sessions for 7 days, but low-certainty evidence warrants further high-quality research
Fang 2025	MA	RCTs	18/18	China, India	ICU patients > 18 years of age	2717	Care bundles plus standard care	Incidence of delirium, delirium duration, hospital length of stay, mortality	Cochrane RoB1; Overall risk of bias judgement by study = HIGH	NR	None	Care bundles may reduce ICU delirium, but heterogeneity limits certainty; large multicenter RCTs are needed to identify optimal components
Formenti 2023	SR	RCTs	12/12	NR	critically ill, adult patients in a medical or surgical ICU	NR	Acupuncture	Incidence of delirium	PEDro; Overall risk of bias judgement by study = MODERATE	NR	None	Acupuncture seems an effective part of a non-pharmacological strategy to manage agitation, pain and delirium in the ICU
Herling 2019	MA (Cochrane)	RCTs	12/4	NR	adult ICU participants aged 18 years and above	1091	Physical or cognitive therapy or both, noise and light reduction in the ICU, and preventive nursing care	Delirium occurrence	Cochrane RoB2; LOW	Moderate to very low quality of evidence	National Institute on Aging	More research is needed to assess the effectiveness of physical and cognitive therapy in preventing ICU delirium, as well as the impact of sedation, environmental changes, and tailored nursing care
Hu 2015	MA (Cochrane)	RCTs	30/3	China, Korea, Japan, Europe, and US	Critically ill adult patients with stable haemodynamic status who were admitted to ICUs or critical care units and had a length of stay of more than 24 h	NR	Earplugs and eye masks, relaxation therapy, sleep-inducing music, massage, foot baths, aromatherapy, valerian acupressure, sound masking, and changing the visiting times of family members	Incidence of delirium	Cochrane RoB2; LOW	Low or very low quality of evidence	School of Nursing, Fujian Medical University, China, National Natural Science Funds of China	Evidence on non-pharmacological sleep interventions in ICU adults is low to very low. Earplugs/eye masks may improve sleep and delirium incidence, but more high-quality research is needed
Jarman 2023	MA	Mixed	12/10	Australia, USA, Iran, France, Turkey, Australia, Germany and UK	Participants aged over 18 admitted to ICU	1460	Physical activity intervention	Incidence of delirium, delirium prevalence, duration of delirium, delirium severity	Cochrane RoB2, PEDro scores and ROBINS-I; Overall risk of bias judgement by study = MODERATE TO HIGH	GRADE: Low quality of evidence	Health Education England (HEE)/National Institute for Health Research (NIHR) and the NIHR Oxford Biomedical Research Centre.	Evidence is insufficient to recommend physical activity alone for ICU delirium; intensity may matter, but more high-quality studies are needed
Kang 2018	MA	Mixed	35/11	NR	Adult ICU patients (age ≥ 18 years)	NR	Multicomponent, physical environment, exercise, patient education, family participation	Incidence of delirium	EPOC; LOW	NR	Basic Science Program through the National Research Foundation of Korea (NRF) funded by the Ministry of Science and ICT	Multicomponent and physical environment interventions were the most widely used methods. Nonpharmacological interventions for the purpose of preventing ICU delirium were effective in reducing the duration of delirium and the delirium occurrence, but not the ICU length of stay and ICU mortality. None of the studies reported adverse reactions related to nonpharmacological interventions
Kang 2023	MA	Mixed	118/59	NR	Adult patients admitted to the ICU	36,356	Non-pharmacological interventions	Incidence of delirium, delirium duration	Cochrane RoB2, ROBIS-I; Overall risk of bias judgement by study = LOW TO HIGH	NR	Korea government and Ministry of Health & Welfare, Republic of Korea	Nonpharmacological interventions improve sleep and help prevent delirium in ICU patients. ICU nurses should adopt interventions that enhance person–environment compatibility to support better outcomes
Li 2025a	NMA	RCTs	17/17	NR	Postoperative adult patients (≥ 18 years) in the ICU	1535	Sleep interventions	Incidence of delirium, ICU length of stay	Cochrane RoB2; Overall risk of bias judgement by study = MODERATE	CINeMA (Confidence in Network Meta-Analysis): Very low quality of evidence	NR	Our study shows that multicomponent non-pharmacological sleep interventions reduce delirium incidence and that circadian rhythm regulation improves sleep quality in postoperative ICU patients. ICU staff should prioritize these strategies to prevent delirium and enhance recovery
Li 2025b	MA	RCTs	11/11	China, Brazil, Iran, Chile, Turkey, and Colombia	Critically ill patients without delirium (≥ 18 years old)	3113	Family involvement	Incidence of delirium, duration of delirium, ICU length of stay	Cochrane RoB2; Overall risk of bias judgement by study = MODERATE	GRADE: Very low to moderate quality of evidence	The National Key Research and Development Program of China	Family involvement plays a key role in preventing delirium in critically ill patients, with direct caregiving participation showing the greatest impact on reducing its incidence
Liang 2021	MA	Mixed	34/10	Asia, US, UK, and Europe	ICU patients > 18 years of age	7159	Multicomponent interventions, early mobilization, family participation, music, patient education, the physical environment, and sleep promotion	Incidence of delirium, duration of delirium, short term mortality, ICU LOS	Cochrane RoB2; LOW	Moderate to low/very low quality of evidence	None	Healthcare professionals should use single or multicomponent interventions (e.g., early mobilization, family participation) to prevent ICU delirium. Further research is needed on patient psychological and family outcomes
Litton 2016	MA	Mixed	9/5	NR	Adult ICU patients	1455	Earplugs and co interventions such as eye mask, relaxing music	Duration of delirium, mortality	Cochrane RoB2; High	NR	NR	Earplugs in ICU patients, alone or with sleep hygiene measures, significantly reduce delirium risk, but the role of co-interventions and optimal sleep strategies remains unclear
Lu 2019	MA	RCTs	13/3	NR	Patients were 18 years of age	94	Bright light	Incidence of delirium	Cochrane RoB2; HIGH	NR	Natural Science Foundation of China ((No. 81502182 and No. 81400893) and CPLA Youth Training Project for Medical Science, China (17QNP030)	Postoperative timed bright light exposure may be helpful for preventing delirium
LV 2025	MA	RCTs	11/11	France, China, UK, Australia, Iran, Ireland	Adult patients admitted to ICU	3352	Patient and Family-Centered Care Interventions	Delirium prevalence, ICU length of stay, depression, anxiety	Cochrane RoB2; Overall risk of bias judgement by study = HIGH	GRADE: Low quality of evidence	NR	PFCC interventions may significantly reduce delirium rates among ICU patients; however, their effects on other outcomes, such as depression, anxiety, and length of stay, were not statistically significant
Matsuura 2023	NMA	Mixed	11/3	Chile, UK, USA, China, Korea, Denmark	Patients admitted to the ICU aged 18 years and above	2549	Nonpharmacological multicomponent interventions	Incidence of delirium	Cochrane RoB2 and ROBINS-I; Overall risk of bias judgement by study = LOW to HIGH	NR	JSPS KAKENHI	Non-pharmacological interventions, especially multicomponent approaches (SP-CS-EM-PC-AS and SP-CS), were most effective in preventing delirium in critically ill patients
Nydahl 2023	MA	RCTs	13/7	USA, Australia, Kore, Germany, Turkey, Chile, Japan	Critically ill adult patients	2164	Early mobilization	Delirium prevention, duration of delirium	JBI; LOW to MODERATE	Moderate quality of evidence	NR	The effect of mobilization on delirium is uncertain, though early mobilization may help prevent and shorten its duration. Further research is needed to determine the optimal approach
Qin 2022	MA	Mixed	6/4	NR	Adult ICU patients	1825	Family intervention	Risk of delirium, duration of delirium, short term mortality, ICU LOS	Cochrane LOW	Moderate quality of evidence	CAMS Innovation Fund for Medical Sciences (CIFMS)	Family intervention can reduce the risk of delirium in ICU patients. Further research is needed to identify specific family intervention therapies, how they are delivered, and their optimal duration
Ryu 2022	MA	Mixed	11/1	NR	ICU patients aged over 19 years	NR	Preventing nursing intervention	Delirium occurrence, ICU LOS	ROBANS 2 for non-RCTs; HIGH	NR	Jungwon University Research Grant	For management of delirium among ICU patients, multi-component intervention packages, suitable for care setting in ICUs, need to be considered for the preparation of nursing intervention for prevention of delirium applicable to nursing practices
Sosnowski 2023	MA	Mixed	18/2	USA, Korea, Italy, India, UK, Australia	Critically ill adult (age ≥ 18 years) patients	29,576	ABCDE/ABCDEF bundle	Incidence of delirium, duration of delirium	JBI; LOW	Low quality of evidence	None	Data from eight studies showed that the ABCDE/F bundle significantly reduced delirium incidence and/or duration in adult ICU patients. However, high heterogeneity and low evidence certainty warrant cautious interpretation
Soylemez 2023	MA	RCTs	14/8	USA, Brazil, UK, Iran, Turkey, Sweden	People with delirium hospitalised in ICU	1123	Multi-sensory stimulation, participation in activities of daily living and family involvement, music therapy, ABCDE package, landscaping and family photo show	Duration of delirium	NR	NR	NR	Nonpharmacological interventions by nurses reduce ICU delirium duration, with effect size influenced by intervention type and study location. This study highlights their effectiveness in ICU care
Teng 2023	MA	RCTs	15/8	NR	ICU patients (age ≥ 18 years)	NR	Sleep interventions (e.g., light therapy, earplugs, melatonin, and multicomponent nonpharmacologic treatments)	Incidence of delirium, ICU length of stay	Cochrane RoB2; Overall risk of bias judgement by study = High	NR	None	The current evidence suggests that non-pharmacological sleep interventions are not effective in preventing delirium in ICU patients
Wang 2020	MA	RCTs	39/5	Germany, China	adult (age > 18 years) ICU patients	774	Early mobilization and rehabilitation (including a range of active or passive physical exercises, except for exclusively neuromuscular electrical stimulation, chest physical therapy, and Chinese medicine acupuncture)	Delirium rate	Cochrane RoB2; HIGH	NR	None	Evidence suggests early mobilization improves muscle strength, reduces ICU complications, and shortens mechanical ventilation and hospital stays. Its impact on delirium, mortality, and post-discharge quality of life requires further large-scale trials
Xu 2022	MA	RCTs	7/7	NR	adult ICU patients with delirium	1291	Cognitive exercise	Incidence of delirium, duration of delirium, hospital LOS	Cochrane RoB2; MODERATE	NR	None	Meta-analysis confirms cognitive exercises shorten ICU delirium duration and hospital stay but do not affect incidence. The lack of post-discharge studies underscores the need for long-term research
Xu 2025	MA	RCTs	14/14	USA, Japan, China, France and Korea	Critically ill patients aged 18 years or older	2151	Sensory-based intervention	Incidence of delirium	Cochrane RoB2; Overall risk of bias judgement by study = HIGH	GRADE: Moderate quality of evidence	Care Fund of the Second Hospital of Shanxi Medical University	Sensory-based interventions reduce delirium in critically ill patients, with auditory interventions showing the greatest benefit
Zhang 2022	MA	Mixed	11/7	NR	Adult (18 years old or older) ICU patients	26,384	ABCDEF bundle	Delirium prevalence, duration of delirium, mortality, ICU LOS, hospital LOS	Modified Jadad Scale and New Castle-Ottawa Scale; LOW	NR	NR	The meta-analysis found no evidence that bundle interventions reduce ICU delirium prevalence or duration but confirmed benefits in lowering coma days, hospital LOS, and 28-day mortality. Many studies did not fully address modifiable delirium risk factors, possibly limiting effectiveness. Rigorous RCTs and full ABCDEF bundle implementation are needed to assess their impact on delirium and related outcomes
Zhao 2024	MA	RCTs	4/4	USA, Chile, Canada	Patients 18 years of age and older who were hospitalized for more than 24 h in the ICU	379	Occupational therapy	Incidence of delirium, duration of delirium, mortality, ICU and hospital length of stay	Cochrane RoB2; Overall risk of bias judgement by study = LOW to HIGH	NR	General Scientific Research Project of Zhejiang Education Department	While occupational therapy is less effective in preventing delirium, it significantly improves ADL and cognitive function in critically ill patients, making it a valuable part of multidisciplinary care
Zhou 2025	MA	Mixed	17/7	NR	critically ill patients or those admitted to the ICU	1794	Early mobilization intervention	Incidence of delirium, duration of delirium, mortality, ICU and hospital length of stay	Cochrane RoB2 and ROBINS-I; Overall risk of bias judgement by study = LOW to MODERATE	NR	None	Early mobilization may reduce delirium risk and duration in critically ill patients and holds promise as a preventive strategy in ICU care

ABCDEF, Assess, Prevent, and Manage Pain, Both Spontaneous Awakening Trials (SAT) and Spontaneous Breathing Trials (SBT), Choice of analgesia and sedation, Delirium: Assess, Prevent, and Manage, Early mobility and Exercise, and Family engagement and empowerment; ICU, Intensive Care Unit; LOS, Length of stay; MA, Meta-analysis; N, Number; NMA, Network Meta-analysis; NR, Not reported; RCT, Randomized controlled trial; RoB, Risk of bias

The correct Table 1:

**Table 1:** Characteristics of included studies

Author year	Review type	Included study design(s)	*N* of included studies / *N* of included non-pharmacological RCTs	Countries	Inclusion criteria	*N* of participants	Review interventions	Outcomes	RoB	Quality/Certainty of the evidence assessment	Funding	Findings
Al Qadheeb 2014	MA	RCTs	17/1	NR	adults (≥19 years or older) admitted to an ICU	49	Early mobilization after daily sedation interruption	Duration of delirium, short term mortality	Cochrane RoB2; LOW	NR	National Center for Advancing Translational Sciences, National Institutes of Health	A review of current evidence fails to support that ICU interventions that reduce delirium duration reduce short-term mortality. Larger controlled studies are needed to establish this relationship
Bannon 2018	MA	RCTs	15/15	USA, Japan, Italy, Canada, Belgium, Netherlands, Chile, UK, Turkey, Thailand and Korea	Critically ill adult patients	2812	Physical and physical with occupational therapy; bright light therapy, range of motion exercises, earplugs, multicomponent orientation and cognitive stimulation protocol, multicomponent occupational therapy including positioning, cognitive training, relative involvement, a mirrors intervention, multicomponent targeting risk factors for delirium, reorientation using family voice	Incidence of delirium, duration of delirium, mortality, sleep quality, cognitive function, quality of life	Cochrane RoB2; LOW	Low quality of evidence	Public Health Agency	Current evidence does not support the use of non-pharmacological interventions in reducing incidence and duration of delirium in critically ill patients. Future research should consider well-designed and well described multicomponent interventions and include adequately defined outcome measures
Burry 2021	MA	RCTs	80/25	North America, Europe and Asia	critically ill adults (≥ 16 years of age in an ICU of any type or high-acuity unit)	NR	Multicomponent, environment physical therapy-occupational therapy	Delirium occurrence, mortality, ICU LOS, hospital LOS	Cochrane RoB2; LOW	NR	Canadian Institutes of Health Research	Single and multi-component non-pharmacological interventions did not connect to any evidence networks to allow for ranking and comparisons as planned; pairwise comparisons did not detect differences compared to standard care
Chen 2022	NMA	RCTs	29/29	Brazil, Denmark, and US	adults (age ≥18 years) in ICU	6571	Light therapy, earplugs, combination of eye mask and earplugs, combination of eye mask, earplugs and melatonin, early mobilization, family visit, reorientation, preoperative patient education and music	Incidence of delirium, duration of delirium	Cochrane RoB2; LOW	NR	Ministry of Science and Technology, Taiwan and Taipei Medical University Shuang Ho Hospital	Multicomponent strategy was the most effective non- pharmacological intervention in reducing the incidence of ICU delirium. Early mobilization and family participation involvement in non-pharmacological interventions seemed to be more effective in reducing the incidence of ICU delirium. These results of network-meta-analysis could be important evidence-based for clinical healthcare providers to optimize the critical care protocol
Dai 2025	MA	RCTs	14/14	Turkey, Iran, America, Korea, China, Netherlands	Patients aged 18 years or older receiving care in ICUs	1434	Music intervention	Risk of delirium	Cochrane RoB1; Overall risk of bias judgement by study = MODERATE	NR	Ministry of Science and Technology of Taiwan	Music interventions may reduce ICU delirium, particularly with two 30-minute daily sessions for 7 days, but low-certainty evidence warrants further high-quality research
Fang 2025	MA	RCTs	18/18	China, India	ICU patients > 18 years of age	2717	Care bundles plus standard care	Incidence of delirium, delirium duration, hospital length of stay, mortality	Cochrane RoB1; Overall risk of bias judgement by study = HIGH	NR	None	Care bundles may reduce ICU delirium, but heterogeneity limits certainty; large multicenter RCTs are needed to identify optimal components
Formenti 2023	SR	RCTs	12/12	NR	critically ill, adult patients in a medical or surgical ICU	NR	Acupuncture	Incidence of delirium	PEDro; Overall risk of bias judgement by study = MODERATE	NR	None	Acupuncture seems an effective part of a non-pharmacological strategy to manage agitation, pain and delirium in the ICU
Herling 2019	MA (Cochrane)	RCTs	12/4	NR	adult ICU participants aged 18 years and above	1091	Physical or cognitive therapy or both, noise and light reduction in the ICU, and preventive nursing care	Delirium occurrence	Cochrane RoB2; LOW	Moderate to very low quality of evidence	National Institute on Aging	More research is needed to assess the effectiveness of physical and cognitive therapy in preventing ICU delirium, as well as the impact of sedation, environmental changes, and tailored nursing care
Hu 2015	MA (Cochrane)	RCTs	30/3	China, Korea, Japan, Europe, and US	Critically ill adult patients with stable haemodynamic status who were admitted to ICUs or critical care units and had a length of stay of more than 24 h	NR	Earplugs and eye masks, relaxation therapy, sleep-inducing music, massage, foot baths, aromatherapy, valerian acupressure, sound masking, and changing the visiting times of family members	Incidence of delirium	Cochrane RoB2; LOW	Low or very low quality of evidence	School of Nursing, Fujian Medical University, China, National Natural Science Funds of China	Evidence on non-pharmacological sleep interventions in ICU adults is low to very low. Earplugs/eye masks may improve sleep and delirium incidence, but more high-quality research is needed
Jarman 2023	MA	Mixed	12/10	Australia, USA, Iran, France, Turkey, Australia, Germany and UK	Participants aged over 18 admitted to ICU	1460	Physical activity intervention	Incidence of delirium, delirium prevalence, duration of delirium, delirium severity	Cochrane RoB2, PEDro scores and ROBINS-I; Overall risk of bias judgement by study = MODERATE TO HIGH	GRADE: Low quality of evidence	Health Education England (HEE)/National Institute for Health Research (NIHR) and the NIHR Oxford Biomedical Research Centre.	Evidence is insufficient to recommend physical activity alone for ICU delirium; intensity may matter, but more high-quality studies are needed
Kang 2018	MA	Mixed	35/11	NR	Adult ICU patients (age ≥ 18 years)	NR	Multicomponent, physical environment, exercise, patient education, family participation	Incidence of delirium	EPOC; LOW	NR	Basic Science Program through the National Research Foundation of Korea (NRF) funded by the Ministry of Science and ICT	Multicomponent and physical environment interventions were the most widely used methods. Nonpharmacological interventions for the purpose of preventing ICU delirium were effective in reducing the duration of delirium and the delirium occurrence, but not the ICU length of stay and ICU mortality. None of the studies reported adverse reactions related to nonpharmacological interventions
Kang 2023	MA	Mixed	118/59	NR	Adult patients admitted to the ICU	36,356	Non-pharmacological interventions	Incidence of delirium, delirium duration	Cochrane RoB2, ROBIS-I; Overall risk of bias judgement by study = LOW TO HIGH	NR	Korea government and Ministry of Health & Welfare, Republic of Korea	Nonpharmacological interventions improve sleep and help prevent delirium in ICU patients. ICU nurses should adopt interventions that enhance person–environment compatibility to support better outcomes
Li 2025a	NMA	RCTs	17/17	NR	Postoperative adult patients (≥18 years) in the ICU	1535	Sleep interventions	Incidence of delirium, ICU length of stay	Cochrane RoB2; Overall risk of bias judgement by study = MODERATE	CINeMA (Confidence in Network Meta-Analysis): Very low quality of evidence	NR	Our study shows that multicomponent non-pharmacological sleep interventions reduce delirium incidence and that circadian rhythm regulation improves sleep quality in postoperative ICU patients. ICU staff should prioritize these strategies to prevent delirium and enhance recovery
Li 2025b	MA	RCTs	11/11	China, Brazil, Iran, Chile, Turkey, and Colombia	Critically ill patients without delirium (≥18 years old)	3113	Family involvement	Incidence of delirium, duration of delirium, ICU length of stay	Cochrane RoB2; Overall risk of bias judgement by study = MODERATE	GRADE: Very low to moderate quality of evidence	The National Key Research and Development Program of China	Family involvement plays a key role in preventing delirium in critically ill patients, with direct caregiving participation showing the greatest impact on reducing its incidence
Liang 2021	MA	Mixed	34/10	Asia, US, UK, and Europe	ICU patients >18 years of age	7159	Multicomponent interventions, early mobilization, family participation, music, patient education, the physical environment, and sleep promotion	Incidence of delirium, duration of delirium, short term mortality, ICU LOS	Cochrane RoB2; LOW	Moderate to low/very low quality of evidence	None	Healthcare professionals should use single or multicomponent interventions (e.g., early mobilization, family participation) to prevent ICU delirium. Further research is needed on patient psychological and family outcomes
Litton 2016	MA	Mixed	9/5	NR	Adult ICU patients	1455	Earplugs and co interventions such as eye mask, relaxing music	Duration of delirium, mortality	Cochrane RoB2; High	NR	NR	Earplugs in ICU patients, alone or with sleep hygiene measures, significantly reduce delirium risk, but the role of co-interventions and optimal sleep strategies remains unclear
Lu 2019	MA	RCTs	13/3	NR	Patients were 18 years of age	94	Bright light	Incidence of delirium	Cochrane RoB2; HIGH	NR	Natural Science Foundation of China ((No. 81502182 and No. 81400893) and CPLA Youth Training Project for Medical Science, China (17QNP030)	Postoperative timed bright light exposure may be helpful for preventing delirium
LV 2025	MA	RCTs	11/11	France, China, UK, Australia, Iran, Ireland	Adult patients admitted to ICU	3352	Patient and Family-Centered Care Interventions	Delirium prevalence, ICU length of stay, depression, anxiety	Cochrane RoB2; Overall risk of bias judgement by study = HIGH	GRADE: Low quality of evidence	NR	PFCC interventions may significantly reduce delirium rates among ICU patients; however, their effects on other outcomes, such as depression, anxiety, and length of stay, were not statistically significant
Matsuura 2023	NMA	Mixed	11/3	Chile, UK, USA, China, Korea, Denmark	Patients admitted to the ICU aged 18 years and above	2549	Nonpharmacological multicomponent interventions	Incidence of delirium	Cochrane RoB2 and ROBINS-I; Overall risk of bias judgement by study = LOW to HIGH	NR	JSPS KAKENHI	Non-pharmacological interventions, especially multicomponent approaches (SP-CS-EM-PC-AS and SP-CS), were most effective in preventing delirium in critically ill patients
Nydahl 2023	MA	RCTs	13/7	USA, Australia, Kore, Germany, Turkey, Chile, Japan	Critically ill adult patients	2164	Early mobilization	Delirium prevention, duration of delirium	JBI; LOW to MODERATE	Moderate quality of evidence	NR	The effect of mobilization on delirium is uncertain, though early mobilization may help prevent and shorten its duration. Further research is needed to determine the optimal approach
Qin 2022	MA	Mixed	6/4	NR	Adult ICU patients	1825	Family intervention	Risk of delirium, duration of delirium, short term mortality, ICU LOS	Cochrane LOW	Moderate quality of evidence	CAMS Innovation Fund for Medical Sciences (CIFMS)	Family intervention can reduce the risk of delirium in ICU patients. Further research is needed to identify specific family intervention therapies, how they are delivered, and their optimal duration
Ryu 2022	MA	Mixed	11/1	NR	ICU patients aged over 19 years	NR	Preventing nursing intervention	Delirium occurrence, ICU LOS	ROBANS 2 for non-RCTs; HIGH	NR	Jungwon University Research Grant	For management of delirium among ICU patients, multi-component intervention packages, suitable for care setting in ICUs, need to be considered for the preparation of nursing intervention for prevention of delirium applicable to nursing practices
Sosnowski 2023	MA	Mixed	18/2	USA, Korea, Italy, India, UK, Australia	Critically ill adult (age ≥ 18 years) patients	29,576	ABCDE/ABCDEF bundle	Incidence of delirium, duration of delirium	JBI; LOW	Low quality of evidence	None	Data from eight studies showed that the ABCDE/F bundle significantly reduced delirium incidence and/or duration in adult ICU patients. However, high heterogeneity and low evidence certainty warrant cautious interpretation
Soylemez 2023	MA	RCTs	14/8	USA, Brazil, UK, Iran, Turkey, Sweden	People with delirium hospitalised in ICU	1123	Multi-sensory stimulation, participation in activities of daily living and family involvement, music therapy, ABCDE package, landscaping and family photo show	Duration of delirium	NR	NR	NR	Nonpharmacological interventions by nurses reduce ICU delirium duration, with effect size influenced by intervention type and study location. This study highlights their effectiveness in ICU care
Teng 2023	MA	RCTs	15/8	NR	ICU patients (age ≥ 18 years)	NR	Sleep interventions (e.g., light therapy, earplugs, melatonin, and multicomponent nonpharmacologic treatments)	Incidence of delirium, ICU length of stay	Cochrane RoB2; Overall risk of bias judgement by study = High	NR	None	The current evidence suggests that non-pharmacological sleep interventions are not effective in preventing delirium in ICU patients
Wang 2020	MA	RCTs	39/5	Germany, China	adult (age > 18 years) ICU patients	774	Early mobilization and rehabilitation (including a range of active or passive physical exercises, except for exclusively neuromuscular electrical stimulation, chest physical therapy, and Chinese medicine acupuncture)	Delirium rate	Cochrane RoB2; HIGH	NR	None	Evidence suggests early mobilization improves muscle strength, reduces ICU complications, and shortens mechanical ventilation and hospital stays. Its impact on delirium, mortality, and post-discharge quality of life requires further large-scale trials
Xu 2022	MA	RCTs	7/7	NR	adult ICU patients with delirium	1291	Cognitive exercise	Incidence of delirium, duration of delirium, hospital LOS	Cochrane RoB2; MODERATE	NR	None	Meta-analysis confirms cognitive exercises shorten ICU delirium duration and hospital stay but do not affect incidence. The lack of post-discharge studies underscores the need for long-term research
Xu 2025	MA	RCTs	14/14	USA, Japan, China, France and Korea	Critically ill patients aged 18 years or older	2151	Sensory-based intervention	Incidence of delirium	Cochrane RoB2; Overall risk of bias judgement by study = HIGH	GRADE: Moderate quality of evidence	Care Fund of the Second Hospital of Shanxi Medical University	Sensory-based interventions reduce delirium in critically ill patients, with auditory interventions showing the greatest benefit
Zhang 2022	MA	Mixed	11/7	NR	Adult (18 years old or older) ICU patients	26,384	ABCDEF bundle	Delirium prevalence, duration of delirium, mortality, ICU LOS, hospital LOS	Modified Jadad Scale and New Castle-Ottawa Scale; LOW	NR	NR	The meta-analysis found no evidence that bundle interventions reduce ICU delirium prevalence or duration but confirmed benefits in lowering coma days, hospital LOS, and 28-day mortality. Many studies did not fully address modifiable delirium risk factors, possibly limiting effectiveness. Rigorous RCTs and full ABCDEF bundle implementation are needed to assess their impact on delirium and related outcomes
Zhao 2024	MA	RCTs	4/4	USA, Chile, Canada	Patients 18 years of age and older who were hospitalized for more than 24 h in the ICU	379	Occupational therapy	Incidence of delirium, duration of delirium, mortality, ICU and hospital length of stay	Cochrane RoB2; Overall risk of bias judgement by study = LOW to HIGH	NR	General Scientific Research Project of Zhejiang Education Department	While occupational therapy is less effective in preventing delirium, it significantly improves ADL and cognitive function in critically ill patients, making it a valuable part of multidisciplinary care
Zhou 2025	MA	Mixed	17/7	NR	critically ill patients or those admitted to the ICU	1794	Early mobilization intervention	Incidence of delirium, duration of delirium, mortality, ICU and hospital length of stay	Cochrane RoB2 and ROBINS-I; Overall risk of bias judgement by study = LOW to MODERATE	NR	None	Early mobilization may reduce delirium risk and duration in critically ill patients and holds promise as a preventive strategy in ICU care

ABCDEF, Assess, Prevent, and Manage Pain, Both Spontaneous Awakening Trials (SAT) and Spontaneous Breathing Trials (SBT), Choice of analgesia and sedation, Delirium: Assess, Prevent, and Manage, Early mobility and Exercise, and Family engagement and empowerment; ICU, Intensive Care Unit; LOS, Length of stay; MA, Meta-analysis; N, Number; NMA, Network Meta-analysis; NR, Not reported; PFCC, Patient and Family-Centered Care Interventions; RCT, Randomized controlled trial; RoB, Risk of bias

The incorrect Table 2:

**Table:** **Table 2** Effectiveness of non-pharmacological and multicomponent care bundle interventions for ICU delirium

Intervention	ICU delirium occurrence	Duration of ICU delirium	Mortality	ICU and hospital LOS
**Circadian/Sleep Interventions**				
Bright light therapy	OR 0.89 (95% CI 0.57 to 1.40); four studies; low to high RoB (45)	MD 0.02 (95% CI −1.15 to 1.19) (25)		
Earplugs and eye masks (or their combination)	**OR 0.42 (95% CI 0.22 to 0.80)**; low to high RoB (45)			
**Family interventions**				
Family participation interventions	**RR 0.46 (95% CI**,** 0.31 to 0 69)**; eleven studies (3,113 participants); very low to moderate certainty evidence; moderate RoB (46)	**WMD − 2.18 (95% CI −4.14 to −0.22)**; three studies (46)	OR 0.68 (95% CI 0.22 to 2.09); two studies (31)	**WMD − 1.46 (95% CI −2**,**43 to −0.50)**; seven studies (ICU LOS) WMD 0.24 (95% CI −0.56 to 1.05); two studies (hospital LOS (46)
Landscaping and family photo displays		MD −0.71 (95% CI −2.54 to 1.12); two studies (36)		
Reorientation using family voice recordings	SMD 0.28 (95% CI −0.60 to 1.16); one study (36)			
**Physical/Environmental Interventions**				
Acupuncture	The incidence of delirium was significantly lower in the treatment group than in the control group.			
Early mobilization	**OR 0.53 (95% CI 0.34 to 0.83)**; 13 studies (2,164 participants); moderate certainty of evidence; low to moderate RoB (30)	**MD −1.78 (95% CI −2.73 to −0.83)**; three studies (296 participants) (30)		MD −1.02 (95% CI −2.88 to 0.84); two studies (282 participants) (ICU LOS) (29)
Fluid management	OR 0.81 (95% CI 0.17 to 3.92); low RoB (25)			
Massage therapy	**OR 0.76 (95% CI**,** 0.62 to 0.93)**; three studies (401 participants); moderate certainty evidence; high RoB (50)			
Mirrors intervention		**SMD − 0.47 (95% CI −0.74 to −0.28)**; one study (36)		
Music therapy	**RR 0.49 (95% CI 0.40 to 0.61)**; 12 studies (1,346 participants); low to high RoB (41)			
Preoperative health education	OR 0.61 (95% CI 0.14 to 2.62); low RoB (25)			
**Cognitive Interventions**				
Cognitive exercises	OR 0.43 (95% CI 0.12 to 1.58); moderate RoB (38)	**MD −1.99 (95% CI −3.20 to −0.79)** (38)		**MD −2.10 (95% CI −2.48 to −1.72)** (hospital LOS) (38)
**Multi-Component Care Bundle**				
ABCDEF bundle	**RR 0.57 (95% CI 0.36 to 0.90)**; six studies (2,000 participants); low certainty evidence; low RoB (35)	**MD −1.37 (95% CI −2.61 to −0.13)**; five studies (3,418 participants) (35)	RR 1.01 (95% CI 0.84 to 1.23); (2,954 participants) (39)	MD −1.08 (95% CI −2.16 to 0.00); nine studies (5,184 participants) (ICU LOS) (39) **MD −1.47 (95% CI −2.80 to −0.15)**; five studies (726 participants) (hospital LOS) (39)
Multicomponent non-pharmacological interventions	**OR 0.48 (95% CI 0.34 to 0.69)**; 13 studies (3,172 participants); moderate to low/very low certainty evidence; low RoB (29)	**MD −1.47 (95% CI −2.2 to −0.75)**; seven studies (1,666 participants) (29)	**OR 0.51 (95% CI 0.26 to 0.97)**; two studies (419 participants) (29)	**MD −1.01 (95% CI −1.77 to −0.25)**; 10 studies (2,036 participants) (ICU LOS) (29)
Preventive nursing care	**RR 0.38 (95% CI 0.32 to 0.45)**; 16 studies; high RoB (42)	**WMD − 1.60 (95% CI −1.96 to −1.23)**; 9 studies (42)	RR 0.78 (95% CI 0.44 to 1.40); Three studies (42)	**WMD − 3.19 (95% CI −4.19 to −2.18)**; 10 studies (Hospital LOS) (42)

ABCDEF, Assess, Prevent, and Manage Pain, Both Spontaneous Awakening Trials (SAT) and Spontaneous Breathing Trials (SBT), Choice of analgesia and sedation, Delirium: Assess, Prevent, and Manage, Early mobility and Exercise, and Family engagement and empowerment; CI, Confidence interval; ICU, Intensive Care Unit; LOS, Length of stay; MD, Mean difference; OR, Odds ratio; RoB, Risk of bias; RR, Risk ratio; SMD, Standardized mean difference; WMD, Weighted mean difference

Text in bold shows significant values

The correct Table 2:

**Table 2:** Effectiveness of non-pharmacological and multicomponent care bundle interventions for ICU delirium

Intervention	ICU delirium occurrence	Duration of ICU delirium	Mortality	ICU and hospital LOS
**Circadian/Sleep Interventions**				
Bright light therapy	OR 0.89 (95% CI 0.57 to 1.40); four studies; low to high RoB (45)	MD 0.02 (95% CI −1.15 to 1.19) (25)		
Earplugs and eye masks (or their combination)	**OR 0.42 (95% CI 0.22 to 0.80)**; low to high RoB (45)			
**Family interventions**				
Family participation interventions	**RR 0.46 (95% CI**,** 0.31 to 0 69)**; eleven studies (3,113 participants); very low to moderate certainty evidence; moderate RoB (46)	**WMD −2.18 (95% CI −4.14 to −0.22)**; three studies (46)	OR 0.68 (95% CI 0.22 to 2.09); two studies (31)	**WMD −1.46 (95% CI −2**,**43 to −0.50)**; seven studies (ICU LOS) WMD 0.24 (95% CI −0.56 to 1.05); two studies (hospital LOS (46)
Landscaping and family photo displays		MD −0.71 (95% CI −2.54 to 1.12); two studies (36)		
Reorientation using family voice recordings	SMD 0.28 (95% CI −0.60 to 1.16); one study (36)			
**Physical/Environmental Interventions**				
Acupuncture	The incidence of delirium was significantly lower in the treatment group than in the control group.			
Early mobilization	**OR 0.53 (95% CI 0.34 to 0.83)**; 13 studies (2,164 participants); moderate certainty of evidence; low to moderate RoB (30)	**MD −1.78 (95% CI −2.73 to −0.83)**; three studies (296 participants) (30)		MD −1.02 (95% CI −2.88 to 0.84); two studies (282 participants) (ICU LOS) (29)
Fluid management	OR 0.81 (95% CI 0.17 to 3.92); low RoB (25)			
Massage therapy	**OR 0.76 (95% CI**,** 0.62 to 0.93)**; three studies (401 participants); moderate certainty evidence; high RoB (50)			
Mirrors intervention		**SMD −0.47 (95% CI −0.74 to −0.28)**; one study (36)		
Music therapy	**RR 0.49 (95% CI 0.40 to 0.61)**; 12 studies (1,346 participants); low to high RoB (41)			
Preoperative health education	OR 0.61 (95% CI 0.14 to 2.62); low RoB (25)			
**Cognitive Interventions**				
Cognitive exercises	OR 0.43 (95% CI 0.12 to 1.58); moderate RoB (38)	**MD −1.99 (95% CI −3.20 to −0.79)** (38)		**MD −2.10 (95% CI −2.48 to −1.72)** (hospital LOS) (38)
**Multi-Component Care Bundle**				
ABCDEF bundle	**RR 0.57 (95% CI 0.36 to 0.90)**; six studies (2,000 participants); low certainty evidence; low RoB (35)	**MD −1.37 (95% CI −2.61 to −0.13)**; five studies (3,418 participants) (35)	RR 1.01 (95% CI 0.84 to 1.23); (2,954 participants) (39)	MD −1.08 (95% CI −2.16 to 0.00); nine studies (5,184 participants) (ICU LOS) (39) **MD −1.47 (95% CI −2.80 to −0.15)**; five studies (726 participants) (hospital LOS) (39)
Multicomponent non-pharmacological interventions	**OR 0.48 (95% CI 0.34 to 0.69)**; 13 studies (3,172 participants); moderate to low/very low certainty evidence; low RoB (29)	**MD −1.47 (95% CI −2.2 to −0.75)**; seven studies (1,666 participants) (29)	**OR 0.51 (95% CI 0.26 to 0.97)**; two studies (419 participants) (29)	**MD −1.01 (95% CI −1.77 to −0.25)**; 10 studies (2,036 participants) (ICU LOS) (29)
Preventive nursing care	**RR 0.38 (95% CI 0.32 to 0.45)**; 16 studies; high RoB (42)	**WMD −1.60 (95% CI −1.96 to −1.23)**; 9 studies (42)	RR 0.78 (95% CI 0.44 to 1.40); Three studies (42)	**WMD −3.19 (95% CI −4.19 to −2.18)**; 10 studies (Hospital LOS) (42)

ABCDEF, Assess, Prevent, and Manage Pain, Both Spontaneous Awakening Trials (SAT) and Spontaneous Breathing Trials (SBT), Choice of analgesia and sedation, Delirium: Assess, Prevent, and Manage, Early mobility and Exercise, and Family engagement and empowerment; CI, Confidence interval; ICU, Intensive Care Unit; LOS, Length of stay; MD, Mean difference; OR, Odds ratio; RoB, Risk of bias; RR, Risk ratio; SMD, Standardized mean difference; WMD, Weighted mean difference

Text in bold shows significant values

**The incorrect Discussion paragraph**:

Future research should prioritise standardising intervention protocols and identifying effective implementation strategies. In particular, there is a pressing need for a well-designed randomised controlled trial (RCT) evaluating a multicomponent intervention that incorporates the most promising elements identified in this review, such as early mobility protocols, family engagement strategies, and targeted environmental modifications, alongside those highlighted in the complementary pharmacological review (Part 1) [REF]. Such a rigorous evaluation would provide a stronger evidence base for clinical practice guidelines that is currently lacking.

**The correct Discussion paragraph**:

Future research should prioritise standardising intervention protocols and identifying effective implementation strategies. In particular, there is a pressing need for a well-designed randomised controlled trial (RCT) evaluating a multicomponent intervention that incorporates the most promising elements identified in this review, such as early mobility protocols, family engagement strategies, and targeted environmental modifications, alongside those highlighted in the complementary pharmacological review (Part 1 [69]). Such a rigorous evaluation would provide a stronger evidence base for clinical practice guidelines that is currently lacking.

Author affiliations, main authors included in the institutional group in the HTML version, Abstract Conclusions, Fig. 1; Tables 1 and 2 and the reference (citation) to part 1 has been updated in this correction article and the original article [1] has been corrected.

## References

[1] Kundakci B, Jones K, Booth A, et al. A systematic meta-review of interventions to prevent and manage delirium in the Intensive Care Unit: Part 2– Non-pharmacological and multicomponent interventions. Crit Care. 2025;29:501. 10.1186/s13054-025-05726-8. 41272698 10.1186/s13054-025-05726-8 PMC12639998

[69] L Jones K, Kundakci B, Booth A, et al. A systematic meta-review of interventions to prevent and manage delirium in the Intensive Care Unit: Part 1–Pharmacological interventions. Crit Care. 2025;29. 10.1186/s13054-025-05615-0. 10.1186/s13054-025-05615-0 PMC12751364 41469920

